# The accuracy and quality of image-based artificial intelligence for muscle-invasive bladder cancer prediction

**DOI:** 10.1186/s13244-024-01780-y

**Published:** 2024-08-01

**Authors:** Chunlei He, Hui Xu, Enyu Yuan, Lei Ye, Yuntian Chen, Jin Yao, Bin Song

**Affiliations:** 1 Department of Radiology, West China Hospital, Sichuan University, Chengdu, 610041 China; 2 Department of Radiology, Sanya People’s Hospital, Sanya, Hainan 572000 China

**Keywords:** Magnetic resonance imaging, Urinary bladder neoplasms, Neoplasm staging, Muscle-invasive bladder neoplasms, Artificial intelligence

## Abstract

**Purpose:**

To evaluate the diagnostic performance of image-based artificial intelligence (AI) studies in predicting muscle-invasive bladder cancer (MIBC). (2) To assess the reporting quality and methodological quality of these studies by Checklist for Artificial Intelligence in Medical Imaging (CLAIM), Radiomics Quality Score (RQS), and Prediction model Risk of Bias Assessment Tool (PROBAST).

**Materials and methods:**

We searched Medline, Embase, Web of Science, and The Cochrane Library databases up to October 30, 2023. The eligible studies were evaluated using CLAIM, RQS, and PROBAST. Pooled sensitivity, specificity, and the diagnostic performances of these models for MIBC were also calculated.

**Results:**

Twenty-one studies containing 4256 patients were included, of which 17 studies were employed for the quantitative statistical analysis. The CLAIM study adherence rate ranged from 52.5% to 75%, with a median of 64.1%. The RQS points of each study ranged from 2.78% to 50% points, with a median of 30.56% points. All models were rated as high overall ROB. The pooled area under the curve was 0.85 (95% confidence interval (CI) 0.81–0.88) for computed tomography, 0.92 (95% CI 0.89–0.94) for MRI, 0.89 (95% CI 0.86–0.92) for radiomics and 0.91 (95% CI 0.88–0.93) for deep learning, respectively.

**Conclusion:**

Although AI-powered muscle-invasive bladder cancer-predictive models showed promising performance in the meta-analysis, the reporting quality and the methodological quality were generally low, with a high risk of bias.

**Critical relevance statement:**

Artificial intelligence might improve the management of patients with bladder cancer. Multiple models for muscle-invasive bladder cancer prediction were developed. Quality assessment is needed to promote clinical application.

**Key Points:**

- Image-based artificial intelligence models could aid in the identification of muscle-invasive bladder cancer.
- Current studies had low reporting quality, low methodological quality, and a high risk of bias.
- Future studies could focus on larger sample sizes and more transparent reporting of pathological evaluation, model explanation, and failure and sensitivity analyses.

**Graphical Abstract:**

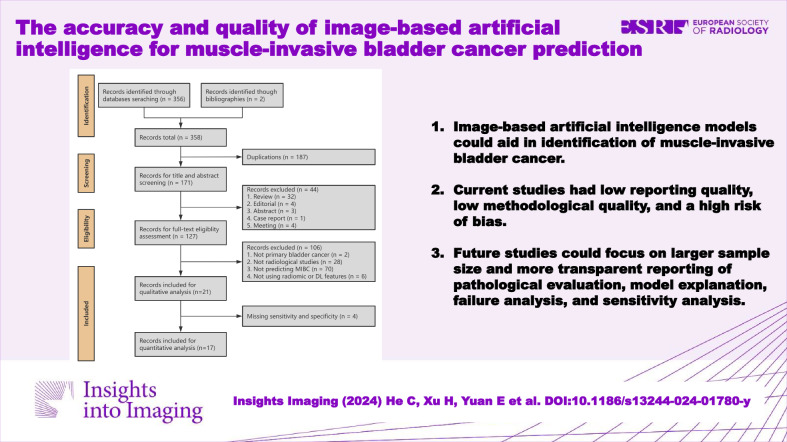

## Introduction

Bladder cancer (BCa) constitutes a significant global health challenge, with an estimated 550,000 new cases and 200,000 deaths worldwide annually [1]. Muscle-invasive bladder cancer (MIBC) is a particularly aggressive form of BCa, defined by the invasion of the tumor into or beyond the superficial muscularis propria of the bladder wall [2]. This subtype is characterized by higher mortality rates, earlier metastasis, and a worse prognosis compared to non-muscle-invasive bladder cancer (NMIBC) [3, 4]. Identifying MIBC promptly is crucial, as it necessitates more aggressive treatments, including radical cystectomy (RC) and adjuvant therapy, which are critical for improving patient outcomes [4, 5].

Clinically, cystoscopy with transurethral resection of bladder tumor (TURBT) is usually the diagnostic approach for identifying MIBC in patients suspected of BCa. While effective, this invasive procedure can occasionally under-sample muscular tissue, resulting in false negative rates of approximately 10% to 15% [6]. The Vesical Imaging-Reporting and Data System (VI-RADS), based on multiparametric magnetic resonance imaging (MRI), has emerged as a valuable non-invasive alternative, offering high sensitivity and specificity in differentiating MIBC from NMIBC [7, 8]. However, the utility of VI-RADS is limited by the long acquisition times, high costs of MRI examinations, and dependence on the subjective experience of the radiologist interpreting the images.

Recent advancements in artificial intelligence (AI), particularly in radiomics and deep learning (DL), provide a promising avenue for the pre-operative identification of MIBC. AI techniques can analyze medical images by extracting hand-crafted radiomic features or using self-learned DL features to predict disease status through sophisticated classification algorithms [9–11]. These technologies have shown potential in enhancing the accuracy and efficiency of MIBC diagnosis [12–32]. Despite the promise, there is a wide variation in the reported results across studies [33, 34]. Furthermore, the overall quality of these studies has not been thoroughly assessed, especially concerning critical methodological aspects such as patient selection, model development, and performance evaluation [33, 34], hindering the clinical application of AI techniques in identifying MIBC.

In this study, we aimed to (1) systematically review and evaluate the diagnostic performances of current AI studies on the prediction of MIBC and (2) use the Checklist for Artificial Intelligence in Medical Imaging (CLAIM), Radiomics Quality Score (RQS), and Prediction model Risk of Bias Assessment Tool (PROBAST) to comprehensively assess the reporting quality and methodological quality of these models [35–37].

## Materials and methods

### Literature search strategy and study selection

The study protocol is registered in the International Prospective Register of Systematic Reviews (CRD42023446035). This systematic review was conducted according to the recommendations published in the Preferred Reporting Items for Systematic Reviews and Meta-Analyses (PRISMA) for Diagnostic Test Accuracy statement [38]. PRISMA checklist is provided in the Supplementary material 1.

Medline via PubMed, Embase via Ovid, Web of Science, and The Cochrane Library were searched for eligible studies from inception to October 30, 2023 using a combination of the following terms: “bladder cancer”, “muscle invasion or staging”, “radiomics or deep learning”, and “computed tomography or magnetic resonance imaging or ultrasound”. The language was restricted to English. The detailed search queries were displayed in Supplementary Material 2. In addition, we screened the bibliographies of initially searched articles for additional relevant studies.

After the removal of duplicated studies, two researchers with 2 and 7 years of experience in genitourinary imaging screened the titles and abstracts of the identified studies. Studies were excluded if the type of the article was one of the following: review, editorial, abstract, and case report. The remaining studies were full-text assessed. To be included, the articles must have fulfilled the following: (1) population: patients with primary BCa; (2) index test: development or validation of radiomics or DL models using computed tomography (CT), MR, or ultrasound images; (3) outcomes: the muscle invasion status confirmed by at least one pathological evaluation method; (4) original articles. Studies were excluded from meta-analysis if they lacked adequate data sufficient to reconstruct the 2 × 2 contingency table.

### Data extraction

Relevant data were extracted from each eligible publication using a standardized form recording the following information: study year, data collection strategy, number of centers, target population, prediction level, sample size, MIBC ratio, gold standard, internal validation method, external validation method, modality, annotation method, number of readers per case, reader agreement, feature extraction method, number of extracted features, number of selected features, and final classifier algorithm. The feature number of DL models was the number of neurons in the first fully-connected layer since the convolutional layers were considered as feature extractors. The following diagnostic accuracy measures were also recorded for meta-analysis: true positive, true negative, false positive, and false negative. When a study involved training and test cohorts, the diagnostic performance in the test cohort was selected for the model’s prediction power; When a study involved external and internal cohorts, the diagnostic performance in the external cohort was selected for the model’s prediction power. If several prediction models were developed in one study, the model with the best performance was chosen.

### CLAIM, RQS, and PROBAST evaluation

The same two researchers independently assessed all eligible publications with CLAIM, RQS, and PROBAST. When a discrepancy occurred, an agreement was reached after discussions with two senior researchers. The consensus data were used in the following analyses. For CLAIM, the study reporting was evaluated by a total of 42 items. The item adherence rate was the percentage of adhering studies over all applicable studies among the item, while the study adherence rate was the percentage of adhering items over all applicable items among the study. The RQS, which consists of 16 criteria, is a recently accepted tool to measure the methodological rigor of radiomics workflow. The total RQS points are the sum of points from checkpoint 1, checkpoint 2, and checkpoint 3, with the ideal RQS points being 100% (36/36.00). For PROBAST, the risk of bias (ROB) was assessed across four domains: participants, predictors, outcome, and analysis. Signaling questions within each domain were answered with one out of five options: “yes, “ “probably yes, “ “probably no, “ “no, “ “no information”. If there was any “no/probably no” in signaling questions, the domain was labeled as having high ROB. If all signaling question is “yes/probably yes”, the domain was labeled as having low ROB. If there was no “no/probably no” but any “no information” in signaling questions, the domain was labeled as having unclear ROB. The overall ROB of the four domains was then determined using the same criteria.

### Statistical analysis

Heterogeneity was evaluated using the following methods: (1) the Cochran *Q* test, with a *p*-value of < 0.05 indicating significant heterogeneity, and (2) the Higgins *I*^2^ test. *I*^2^ values of 0–25%, 25–50%, 50–75%, and > 75% represent very low, low, medium, and high heterogeneity, respectively. The weight of each study was calculated with the inverse variance method, in which the weight given to each study is chosen to be the inverse of the variance of the effect estimate, minimizing the uncertainty of the pooled effect estimate. In the case of medium and high heterogeneity, the random-effect model was favored over the fixed-effect model. Diagnostic accuracy was assessed using the hierarchical summary receiver operating characteristic (HSROC) curves and areas under the HSROC (AUC). Sensitivity, specificity, positive likelihood ratio, and negative likelihood ratio were also calculated. Meta-regression was performed to explore the potential sources of heterogeneity.

All calculations were performed with a 95% confidence interval (95% CI). A difference was considered statistically significant when the *p*-value was smaller than 0.05. We used the “metandi” and “midas” modules in Stata 17 for statistical analyses [39, 40].

## Results

### Characteristics of included studies

**Fig. 1:**
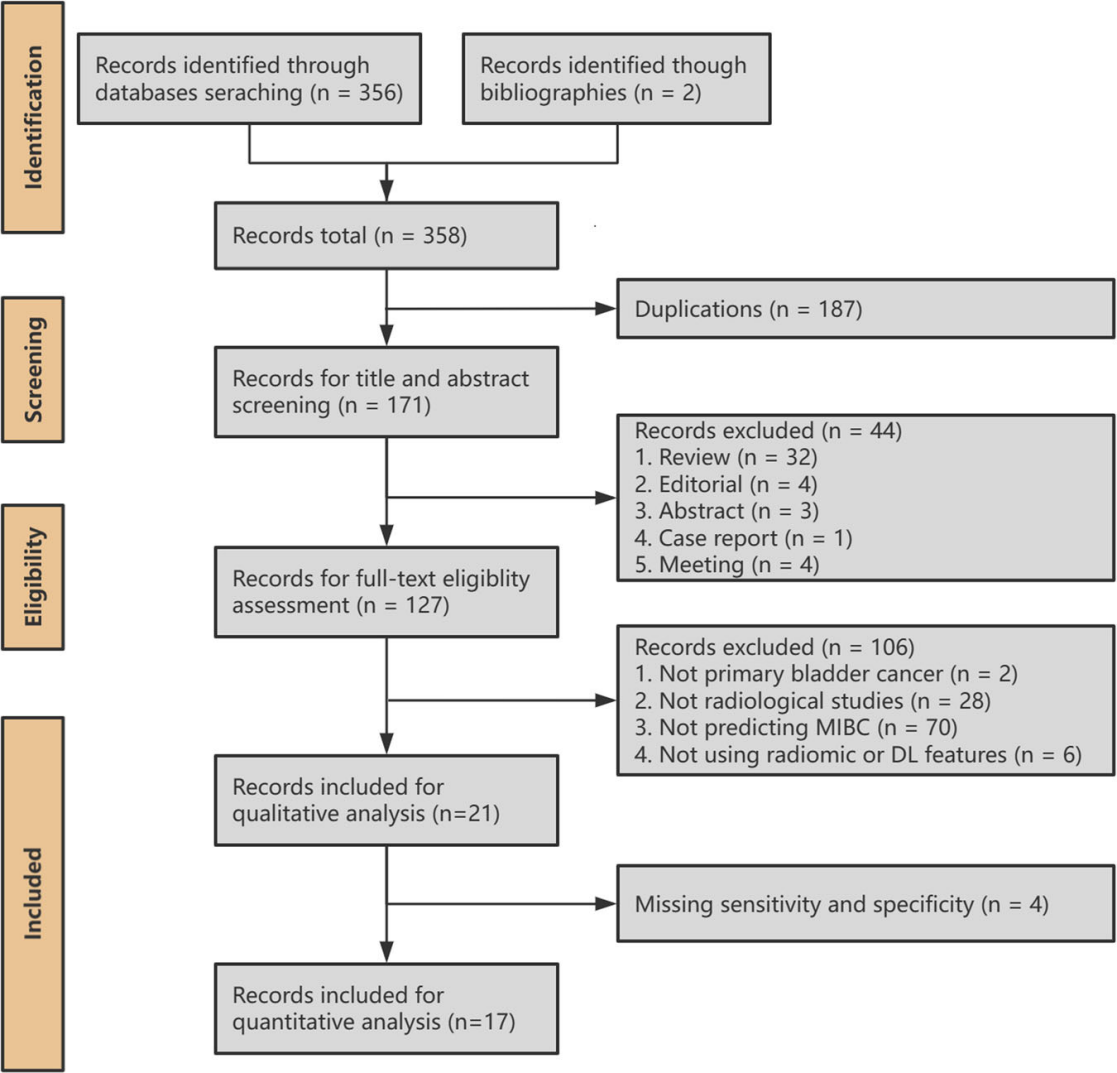
Systematic review flow diagram designed according to PRISMA

**Fig. 2:**
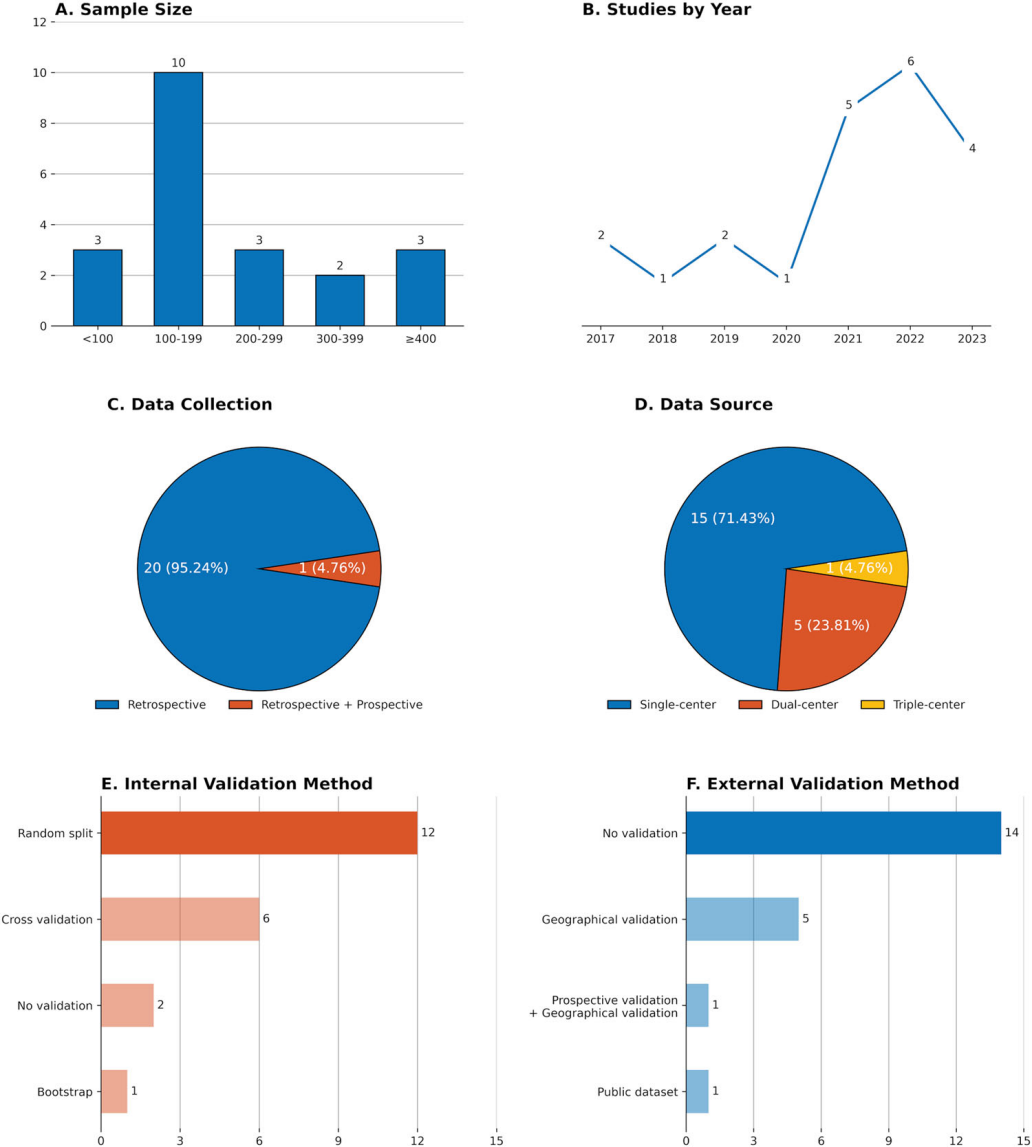
Overview of study characteristics. **A** Aggregate number of patients included in the study; **B** Year of publication; **C** Data collection strategy; **D** Data source; **E** Internal validation method; **F** External validation method

A flowchart depicting the study selection process is shown in Fig. 1. The search strategy identified 171 studies after removing duplicates. Among these, 21 studies met the inclusion criteria. The included studies are summarized in Table 1 and Fig. 2.

**Table 1:** Characteristics of included studies

Citation	Study year	Data collection	Number of centers	Population	Prediction level	Sample size	MIBC ratio	Source of gold standard	Internal validation	External validation
Xu et al [12]	2017	Retrospective	1	BCa	Lesion	118	0.7119	TURBT	No validation	No validation
Garapati et al [13]	2017	Retrospective	1	BCa	Lesion	84	0.4881	Lack information	2-fold cross-validation	No validation
Xu and Zhang et al [14]	2018	Retrospective	1	BCa	Patient	54	0.5556	Surgery	100-run 10-fold cross-validation	No validation
Zheng et al [15]	2019	Retrospective	1	UC	Patient	199	0.4372	RC or TURBT	Bootstrapping	Public dataset
Xu and Yao et al [16]	2019	Retrospective	1	high-grade UC	Patient	218	0.6468	RC	Random split	No validation
Wang et al [17]	2020	Retrospective	2	BCa	Patient	106	0.566	RC, PC, or TURBT	No validation	Geographical validation
Zhou et al [18]	2021	Retrospective	1	BCa	Patient	100	0.5	Surgery	Random split	No validation
Zhang et al [19]	2021	Retrospective	2	UC	Patient	441	0.2766	RC or TURBT	Random split	Geographical validation
Zheng and Xu et al [20]	2021	Retrospective	1	UC	Patient	185	0.3351	Surgery	Random split	No validation
Yang et al [21]	2021	Retrospective	1	UC	Patient	369	0.3252	RC	Random split	No validation
Gao et al [22]	2021	Retrospective	1	BCa	Patient	104	0.4135	Surgery	Random split	No validation
Chen et al [23]	2022	Retrospective	1	UC	Patient	173	0.2486	RC, PC, or TURBT	Random split	No validation
Zou et al [24]	2022	Retrospective + prospective	3	BCa	Patient	468	0.2863	TURBT	Random split	Prospective validation; geographical validation
Zhang and Li et al [25]	2022	Retrospective	1	BCa	Patient	342	0.231	RC or TURBT	Random split	No validation
Cui et al [26]	2022	Retrospective	1	BCa	Patient	188	0.5	RC, PC, or TURBT	Random split	No validation
Zhang and Wu et al [27]	2022	Retrospective	2	BCa	Patient	441	0.2766	RC or TURBT	10-run 10-fold cross-validation	Geographical validation
Liu et al [28]	2022	Retrospective	1	UC	Patient	206	0.2718	Surgery	10-run 5-fold cross-validation	No validation
Sarkar et al [29]	2023	Retrospective	1	UC	Patient	65	0.6307	RC	10-fold cross-validation	No validation
Li et al [30]	2023	Retrospective	2	UC	Lesion	121	0.3305	RC, PC, or TURBT	5-fold cross-validation	Geographical validation
Wang and Li et al [31]	2023	Retrospective	1	UC	Patient	191	0.3717	RC or TURBT	Random split	No validation
Li and Cao et al [32]	2023	Retrospective	2	UC	Lesion	215	0.2884	RC, PC, or TURBT	Random split	Geographical validation

*BCa* bladder cancer, *MIBC* muscle-invasive bladder cancer, *UC* urothelial carcinoma, *TURBT* transurethral resection of bladder tumor, *RC* radical cystectomy, *PC* partial cystectomy

The included 21 studies were published between September 2017 and May 2023, of which approximately two-thirds (15/21, 71.4%) were published within the past three years. Most of the studies (20/21, 95.2%) were retrospectively designed except for one [24]. The population of these studies varied. Ten studies included patients with BCa [12–14, 17, 18, 22, 24–27], one study analyzed patients with high-grade urothelial carcinoma [16], and other studies analyzed patients with urothelial carcinoma [15, 16, 19–21, 23, 28–32]. Four studies performed lesion-level prediction while others performed patient-level prediction [12, 13, 30, 32]. The sample size of the studies ranged from 54 to 468 patients, with a total of 4256 patients and 4388 lesions analyzed in 21 studies. The prevalence of MIBC ranged from 23.1% to 71.2%, with a median of 37.2%. The reference standard for the diagnosis of MIBC also varied. Ten studies diagnosed MIBC by specimens from different surgical techniques of TURBT, RC, or partial cystectomy (PC) [15, 17, 19, 23, 25–27, 30–32]. Three studies only included RC patients [16, 21, 29]. Two studies only included TURBT patients [12, 24]. However, there were still five studies that did not specify the source of the specimen [13, 14, 20, 22, 28]. The most common internal validation method was random-split validation [16, 18–26, 31, 32], while most studies (15/21, 71.4%) did not perform external validation except for six studies [17, 19, 24, 27, 30, 32].

### AI technique details of the included studies

The AI technique details of the included studies are summarized in Table 2. All the studies analyzed a single modality. The most common modality was MRI (*n* = 12), followed by CT (*n* = 8) and ultrasound (*n* = 1). Manual annotation (*n* = 15) was the major method for delineating the region of interest (ROI), while other studies used semi-automatic (*n* = 4) or fully-automatic (*n* = 2) segmentation algorithms. Fourteen studies extracted hand-crafted radiomic features, five studies extracted self-learned DL features [13, 19, 21, 24, 29, 32], and two studies extracted both types of features [23, 30]. Extracted feature counts ranged from 63 to 23,688 in the included studies, and the selected feature counts ranged from 6 to 2048.

**Table 2:** Methodological characteristics of the included studies

Citation	Modality	Annotation	Number of readers per case	Reader agreement after annotation	Feature extraction	Number of extracted features	Number of selected features	Final classifier	AUC
Xu et al [12]	MRI	Manual; 3D	2	Yes	Radiomics	63	30	Support vector machine	/
Garapati et al [13]	CT	Automatic; 3D	1	No	Radiomics	91	Lack information	Random forest	/
Xu and Zhang et al [14]	MRI	Manual; 2D	1	No	Radiomics	1104	19	Support vector machine	0.9857
Zheng et al [15]	MRI	Semi-automatic; 3D	1	Yes	Radiomics	2062	23	LASSO	/
Xu and Yao et al [16]	MRI	Manual; 3D	1	No	Radiomics	156	21	Random forest	0.907
Wang et al [17]	MRI	Manual; 2D	2	Yes	Radiomics	1404	36	Logistic regression	0.672
Zhou et al [18]	CT	Semi-automatic; 3D	2	No	Radiomics	1223	6	Support vector machine; logistic regression	0.782
Zhang et al [19]	CT	Semi-automatic; 3D	2	No	Deep learning	Lack information	Lack information	FGP-Net	0.791
Zheng and Xu et al [20]	MRI	Manual; 3D	1	No	Radiomics	2436	21	LASSO	0.906
Yang et al [21]	CT	Manual; 2D	2	Yes	Deep learning	49	49	Neural network	0.998
Gao et al [22]	US	Manual; 2D	2	Yes	Radiomics	5936	30	Naïve Bayes	0.84
Chen et al [23]	CT	Manual; 3D	Lack information	No	Both	1735; 2048	30	Logistic regression	0.884
Zou et al [24]	MRI	Automatic; 2D	2	Yes	Deep learning	2048	2048	Inception V3	0.856
Zhang and Li et al [25]	MRI	Manual; 3D	2	Yes	Radiomics	23,688	9	Logistic regression	0.931
Cui et al [26]	CT	Manual; 3D	1	No	Radiomics	102	8	AdaBoost	0.894
Zhang and Wu et al [27]	CT	Semi-automatic; 3D	2	No	Radiomics	1218	8	Logistic regression	0.784
Liu et al [28]	MRI	Manual; 2D	1	No	Radiomics	4128	28	LASSO	/
Sarkar et al [29]	CT	Manual; 3D	1	Lack information	Deep learning	2048–9216	Lack information	Linear discriminant analysis	/
Li et al [30]	MRI	Manual; 3D	1	No	Both	851; Lack information	Lack information	Neural network	0.932
Wang and Li et al [31]	MRI	Manual; 3D	2	No	Radiomics	1070	6	Logistic regression	0.711
Li and Cao et al [32]	MRI	Manual; 3D	1	No	Deep learning	2048	2048	ResNet	0.861

*US* ultrasound, *LASSO* least absolute shrinkage and selection operator

### Quality evaluation

**Fig. 3:**
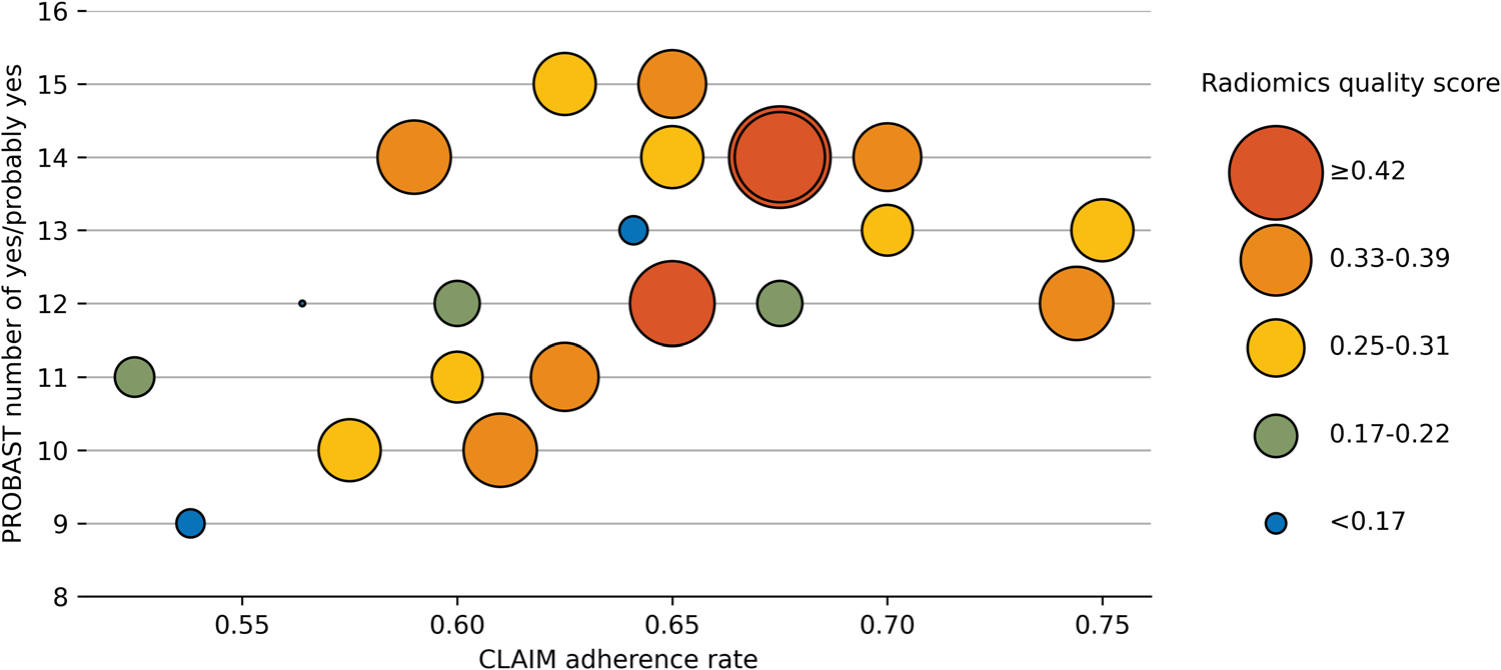
Diagram showing reporting quality, methodological quality, and risk of bias of each study. The *x*-axis is the CLAIM adherence rate. The *y*-axis is the number of yes or probably yes in the PROBAST evaluation. The size of each point is the RQS points

The CLAIM study adherence rates, RQS points, and number of yes/probably yes in PROBAST of each study were shown in Fig. 3.

#### CLAIM

**Fig. 4:**
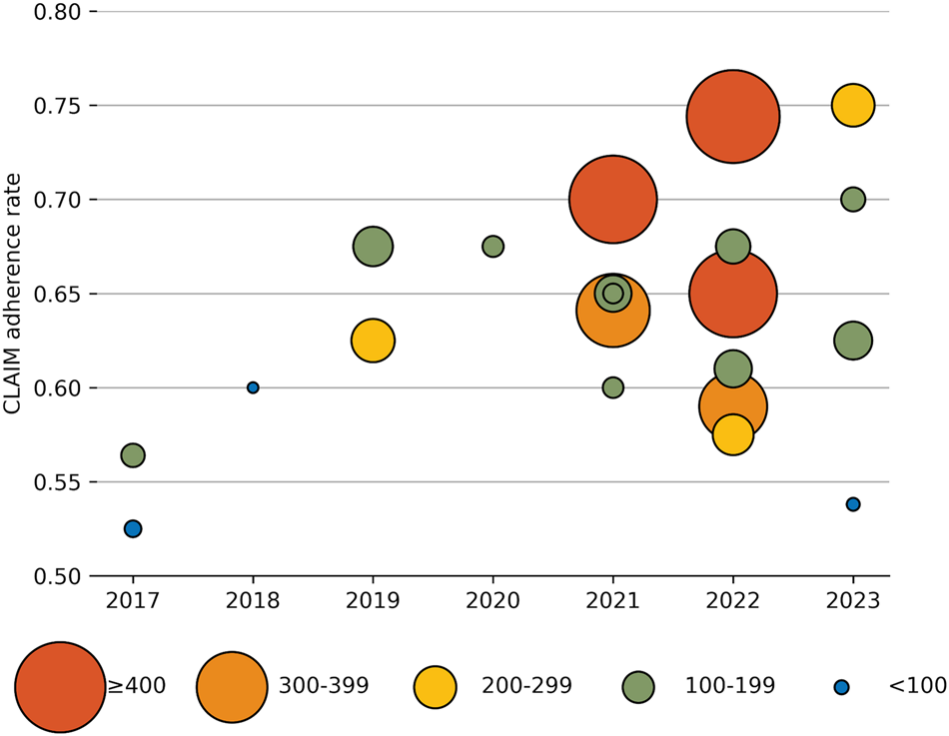
Diagram showing reporting quality by year and sample size. The *x*-axis is the year of publication. The *y*-axis is the CLAIM adherence rate. The size of each point is the sample size

The study adherence rates on CLAIM of each study are shown in Fig. 4. The study adherence rate on CLAIM ranged from 52.5% to 75%, with a median of 64.1%. Five studies had a CLAIM study adherence rate lower than 60.0% [12, 13, 25, 28, 29]. The detailed CLAIM evaluation can be found in Supplementary Material 3.

The item adherence rates for CLAIM are displayed in Table 3. The item adherence rate for CLAIM ranged from 0.0% to 100.0%, with a median of 85.7%. A total of 14 items had poor adherence rates (CLAIM item adherence rate < 60.0%), including data preprocessing (item 9, 42.9%), de-identification (item 12, 0.0%), missing data handling (item 13, 9.5%), alternative reference standard choosing (item 15, 6.3%), pathological evaluation standard (item 16, 4.7%), pathological evaluation method (item 17, 0.0%), pathological evaluation variability measurement (item 18, 0.0%), sample size estimation (item 19, 0.0%), model parameters initialization method (item 24, 14.3%), sensitivity analysis (item 30, 0.0%), explainability or interpretability method (item 31, 23.8%), external data validation (item 32, 33.3%), failure analysis (item 37, 14.3%), and full study protocol (item 41, 0.0%).

**Table 3:** Reporting quality assessment using the CLAIM

Section	Item	Adherence studies	Applicable studies	Adherence rate	Section	Item	Adherence studies	Applicable studies	Adherence rate
Title or Abstract	item_1	21	21	100%	Methods—Model	item_22	20	21	95.24%
	item_2	20	21	95.24%		item_23	18	21	85.71%
Introduction	item_3	21	21	100%		item_24	3	21	14.29%
	item_4	20	21	95.24%	Methods—Training	item_25	21	21	100%
Methods—Study Design	item_5	21	21	100%		item_26	21	21	100%
	item_6	21	21	100%		item_27	0	0	/
Methods—Data	item_7	21	21	100%	Methods—Evaluation	item_28	21	21	100%
	item_8	19	21	90.48%		item_29	18	21	85.71%
	item_9	9	21	42.86%		item_30	0	21	0%
	item_10	1	1	100%		item_31	5	21	23.81%
	item_11	21	21	100%		item_32	7	21	33.33%
	item_12	0	21	0%	Results—Data	item_33	13	21	61.9%
	item_13	2	21	9.52%		item_34	18	21	85.71%
Methods—Ground Truth	item_14	16	21	76.19%	Results—Model performance	item_35	21	21	100%
	item_15	1	16	6.25%		item_36	18	21	85.71%
	item_16	1	21	4.76%		item_37	3	21	14.29%
	item_17	0	21	0%	Discussion	item_38	21	21	100%
	item_18	0	21	0%		item_39	15	21	71.43%
Methods—Data Partitions	item_19	0	21	0%	Other information	item_40	19	21	90.48%
	item_20	21	21	100%		item_41	0	21	0%
	item_21	21	21	100%		item_42	13	21	61.9%

#### RQS

**Fig. 5:**
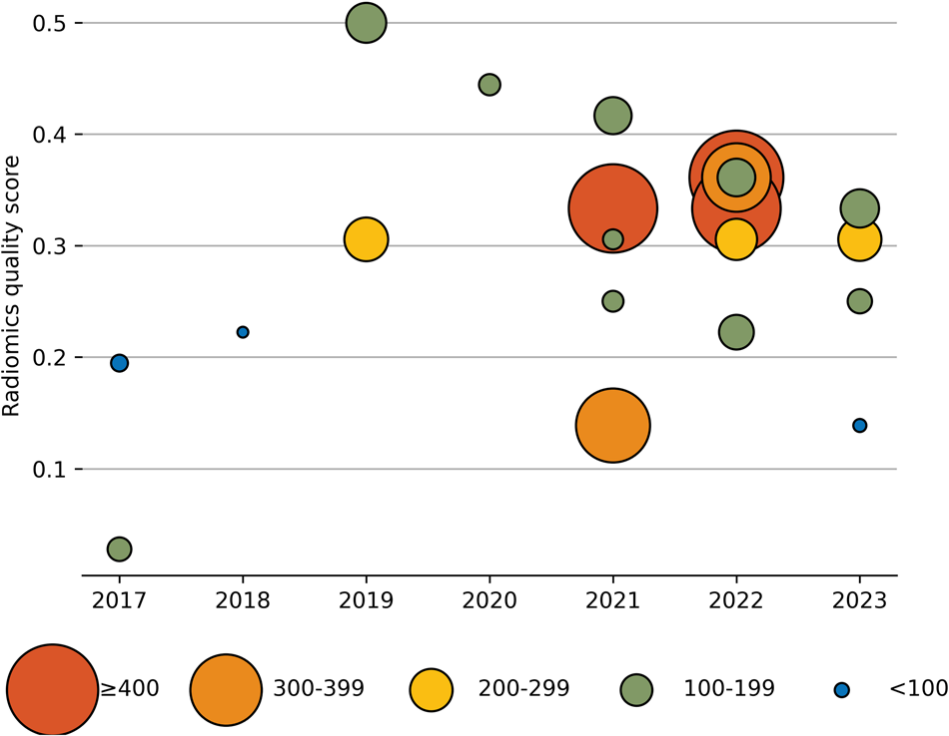
Diagram showing methodological quality by year and sample size. The *x*-axis is the year of publication. The *y*-axis is the RQS points. The size of each point is the sample size

The RQS points of each study are shown in Fig. 5, and the median RQS points for each criterion are displayed in Table 4. The RQS points of each study ranged from 2.78% to 50% points, with a median of 30.56% points. The detailed RQS evaluation and checklist can be found in Supplementary Material 3.

**Table 4:** Methodological quality assessment using the RQS

Criterion	Points range	Median points (percentage)
Image protocol quality	0–2	1 (50%)
Multiple segmentations	0–1	1 (50%)
Phantom study on all scanners	0–1	0 (0%)
Imaging at multiple time points	0–1	0 (0%)
Feature reduction or adjustment for multiple testing	−3 to 3	3 (100%)
Multivariable analysis with non-radiomics features	0–1	0 (0%)
Detect and discuss biological correlates	0–1	0 (0%)
Cut-off analyses	0–1	0 (0%)
Discrimination statistics	0–2	1 (50%)
Calibration statistics	0–2	0 (0%)
Prospective study registered in a trial database	0–7	0 (0%)
Validation	−5 to 5	2 (40%)
Comparison to ‘gold standard'	0–2	0 (0%)
Potential clinical utility	0–2	0 (0%)
Cost-effectiveness analysis	0–1	0 (0%)
Open science and data	0–4	0 (0%)
Total	−8 to 36	11 (30.6%)

Median points percentage was calculated by dividing the scored points by the ideal points

Most of the studies (18/21, 85.7%) had presented their image protocols except for three [13, 18, 29], while no study reported the use of a public protocol. Less than half of the studies (6/21, 28.6%) did not perform multiple segmentations to control the inter- or intra-rater variability of feature extraction [13, 14, 24, 29, 30, 32], no study analyzed inter-scanner differences and temporal variabilities of the features. All studies that used radiomic features and one study that used both radiomic and DL features reduced the dimension of features, while most DL-only studies (4/21, 19.0%) did not perform feature selection on DL features. Seven studies (33.3%) combined clinical information with radiomic models [15–18, 20, 25, 31], and nine (42.9%) compared the radiomic models with radiologist’s diagnosis or VI-RADS category [15–17, 19, 20, 23, 28, 31, 32]. The models performed better than radiologists in internal validation, but their generalizability to external validation data was not as good as experienced radiologists. Only one study discussed the relevance between radiomic features and clinical/histological phenotypes [22]. Most studies (20/21, 95.2%) reported the discrimination statistics except for one [29], but only less than half of the studies (8/21, 38.1%) reported calibration statistics [15, 17–20, 25, 27, 30] as well as (9/21, 42.9%) the cut-off analysis [15, 17, 19, 20, 25–27, 31, 32]. Only one study (4.8%) used prospectively collected data [24]. Only one did not (1/21, 4.8%) perform validation of radiomic signatures [12], and seven (33.3%) externally validated their models on data from other institutes [15, 17, 19, 24, 27, 30, 32]. Only eight studies (38.1%) used decision curve analysis to determine the clinical utility of models [15, 17–20, 25, 26, 30]. Finally, no study conducted cost-effectiveness analyses or shared code or representative data for model development and inference.

#### PROBAST

**Fig. 6:**
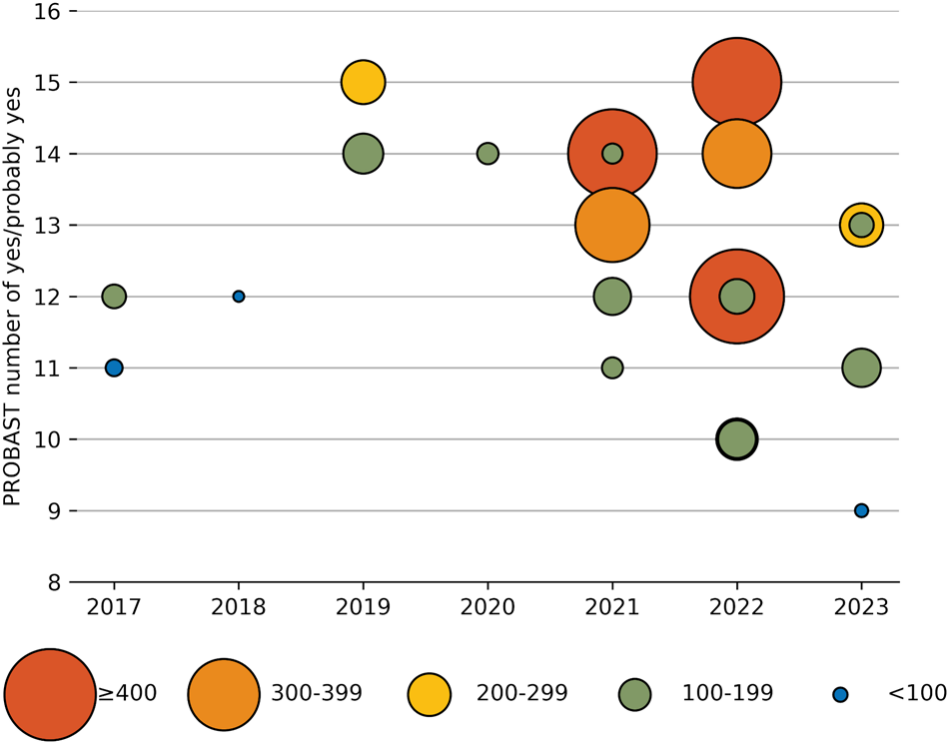
Diagram showing the risk of bias by year and sample size. The *x*-axis is the year of publication. The *y*-axis is the number of yes or probably yes in the PROBAST evaluation. The size of each point is the sample size

The results of the PROBAST evaluation are shown in Fig. 6 and Table 5. In total, all studies were rated as having a high overall risk of bias. Models in two studies were rated as unclear ROB in participants domain due to poor-documented eligibility criteria [13, 29]. In the predictors domain, models in four studies were rated as high ROB [24, 26, 28, 29], and models in ten studies were rated as unclear ROB [14–16, 18, 20, 21, 23, 30–32]. The annotation was done by more than one rater but the inter-rater variability was not analyzed, resulting in probably different predictor definitions for all participants. Blind annotation was the major source of high or unclear ROB. Raters in two studies annotated the image with knowledge of pathological evaluation results [24, 26], and twelve studies did not specify whether the annotation was done blindly [14–16, 18, 20, 21, 23, 28–32]. In the outcome domain, only one study was rated as low ROB [16] while the others were rated as unclear ROB. Most of the studies (20/21) did not report how MIBC was determined in pathological evaluation, how many pathologists were enrolled, and whether the annotation was done blindly. In the analysis domain, all models were rated as having a high overall ROB. Inadequate sample size and poor model performance examination both contributed to the potential high ROB of these AI models in this domain. The detailed PROBAST evaluation can be found in Supplementary Material 3.

**Table 5:** Risk of bias assessment using the PROBAST

Signaling question	Yes or probably yes	No information	No or probably no
Domain 1: participants			
Were appropriate data sources used?	21 (100%)	0 (0%)	0 (0%)
Were all inclusions and exclusions of participants appropriate?	19 (90.48%)	2 (9.52%)	0 (0%)
Domain 2: predictors			
Were predictors defined and assessed in a similar way for all participants?	18 (85.71%)	1 (4.76%)	2 (9.52%)
Were predictor assessments made without knowledge of outcome data?	7 (33.33%)	12 (57.14%)	2 (9.52%)
Are all predictors available at the time the model is intended to be used?	21 (100%)	0 (0%)	0 (0%)
Domain 3: outcome			
Was the outcome determined appropriately?	17 (80.95%)	4 (19.05%)	0 (0%)
Was a prespecified or standard outcome definition used?	1 (4.76%)	20 (95.24%)	0 (0%)
Were predictors excluded from the outcome definition?	21 (100%)	0 (0%)	0 (0%)
Was the outcome defined and determined in a similar way for all participants?	1 (4.76%)	20 (95.24%)	0 (0%)
Was the outcome determined without knowledge of predictor information?	1 (4.76%)	20 (95.24%)	0 (0%)
Was the time interval between predictor assessment and outcome determination appropriate?	17 (80.95%)	4 (19.05%)	0 (0%)
Domain 4: analysis			
Were there a reasonable number of participants with the outcome?	1 (4.76%)	1 (4.76%)	19 (90.48%)
Were continuous and categorical predictors handled appropriately?	21 (100%)	0 (0%)	0 (0%)
Were all enrolled participants included in the analysis?	20 (95.24%)	0 (0%)	1 (4.76%)
Were participants with missing data handled appropriately?	2 (9.52%)	19 (90.48%)	0 (0%)
Was selection of predictors based on univariable analysis avoided?	16 (76.19%)	0 (0%)	5 (23.81%)
Were complexities in the data accounted for appropriately?	21 (100%)	0 (0%)	0 (0%)
Were relevant model performance measures evaluated appropriately?	8 (38.1%)	0 (0%)	13 (61.9%)
Were model overfitting, underfitting, and optimism in model performance accounted for?	7 (33.33%)	0 (0%)	14 (66.67%)
Do predictors and their assigned weights in the final model correspond to the results from the reported multivariable analysis?	21 (100%)	0 (0%)	0 (0%)

### Clinical value of AI in predicting MIBC

Four studies were excluded for not providing detailed information about sensitivity and specificity in the meta-analysis [12, 13, 15, 28]. A total of seventeen studies were included in the meta-analysis. Seven studies evaluated the diagnostic performance using CT [18, 19, 21, 23, 26, 27, 29], nine using MRI [14, 16, 17, 20, 24, 25, 30–32] and one using ultrasound [22]. The pooled sensitivity, specificity, and AUC for CT were 0.82 (95% CI 0.72–0.89), 0.79 (95% CI 0.72–0.84), and 0.85 (95% CI 0.81–0.88). For MRI, the pooled sensitivity, specificity and AUC were 0.84 (95% CI 0.75–0.91), 0.79 (95% CI 0.77–0.92), and 0.92 (95% CI 0.89–0.94). Ten studies assessed MIBC prediction using radiomics [14, 16–18, 22, 25–27, 31], six using deep learning [19, 21, 24, 29, 30, 32] and one using both [23]. The pooled sensitivity, specificity, and AUC for radiomics were 0.84 (95% CI 0.76–0.90), 0.82 (95% CI 0.74–0.87), and 0.89 (95% CI 0.86–0.92). In terms of deep learning, the pooled sensitivity, specificity and AUC were 0.81 (95% CI 0.68–0.89), 0.87 (95% CI 0.74–0.94), and 0.91 (95% CI 0.88–0.93). The forest plots and AUC curves are illustrated in Supplementary Material 2.

## Discussion

AI techniques have been widely studied in MIBC identification. Our systematic review comprehensively evaluated the reporting quality, methodological quality, and ROB of current AI studies for MIBC prediction. The results showed that the overall quality of these studies was poor, with a median CLAIM study adherence rate of 64.1%, a median RQS points percentage of 30.6%, and a high ROB among all studies. The meta-analysis showed current MIBC-predictive AI models had good performance with an AUC of 0.85 (95% CI 0.81–0.88) for CT, 0.92 (95% CI 0.89–0.94) for MRI, 0.89 (95% CI 0.86–0.92) for radiomics and 0.91 (95% CI 0.88–0.93) for deep learning. The current results indicate that AI models have a high potential for predicting MIBC but are far from useful tools in clinical practice.

Two systematic reviews have previously evaluated radiomic studies for MIBC prediction using RQS [33, 34]. Most of the RQS results in our study were similar to theirs. However, a difference in the “Comparison to Gold standard” part was observed. In the previous reviews, most studies were assigned two points for comparing the models with the current gold standard. In our review, less than half of the studies were assigned two points. To show the added value of radiomics, we believe that the “gold standard” refers to the commonly-used non-invasive methods in current clinical practice for detecting MIBC (i.e., manual image interpretation with or without VI-RADS category) [4], thus we only assigned two points to nine studies that had compared the models with manual interpretations. The results of the nine studies showed the AI models usually performed better than radiologists in internal validation, but their generalizability to external validation data was not as good as experienced radiologists [15–17, 19, 20, 26, 28, 31, 32].

Using CLAIM and PROBAST, our systematic review identified some unique quality-reducing items in MIBC-predictive AI studies. Firstly, for the pathology gold standard, only one study reported how MIBC was confirmed through histopathological investigation [16]. Most studies only reported the source of the specimen, and none of the studies reported the details of the histopathological investigation, including the number of pathologists, the inter-reader agreement, and the blindness of assessment. The results of the meta-analysis showed different pathological gold standards significantly contributed to the heterogeneity of sensitivity and specificity. About half of the studies used multiple surgical techniques to obtain the specimen, however, none of them reported the criteria for choosing the reference standard for individual patients or compared the model performance in patients who underwent TURBT with that in patients who underwent RC or PC. Secondly, statistical concerns were poorly considered in current studies. No study calculated the minimal sample size. When internally validating the model, only a few studies avoided over-pessimistic or over-optimistic evaluation of model performance by using cross-validation or bootstrapping. Many studies used cross-validation to select the best hyperparameter or the best model in the training set. However, nested cross-validation is needed to evaluate the model performance while selecting optimal hyperparameters [41]. In addition, only a few studies evaluated the calibration of AI models. Thirdly, current studies lacked analyses beyond performance evaluation. The explainability was poorly discussed, especially in radiomic studies, and few studies performed failure analysis or sensitivity analysis. Finally, no study reported the de-identification method for the clinical, pathological, and image data. Blind assessment during the pathological and radiological evaluation was also poorly reported.

There are some limitations in this study. First, only a subset of eligible studies met the selection criteria for meta-analyses, and significant heterogeneity existed among studies, thus it is important to interpret meta-analysis results with caution. Second, we only used RQS, PROBAST, and CLAIM in the evaluations. The quality of radiomics has always been a hot topic, which affects the repeatability and reproducibility of radiomics and limits its widespread clinical application. Therefore, various checklists and tools have been proposed for quality evaluation. Recently, the CheckList for EvaluAtion of Radiomics research (CLEAR) and METhodological RadiomICs Score (METRICS) were proposed and were regarded as better alternatives to CLAIM and RQS for radiomic studies [42, 43]. However, our studies did not include these newly developed tools. Thirdly, CLAIM, RQS, and PROBAST contain some elements of subjective judgment, and borderline results may impact overall interpretation.

In conclusion, although AI techniques show high diagnostic performance in predicting MIBC, the insufficient quality of studies suggests that these AI models are not currently available for clinical use. Future studies could focus on more transparent reporting of pathological evaluation, larger sample size, and additional analyses, such as prediction explanation, failure analysis, and sensitivity analysis.

### Supplementary Information

ELECTRONIC SUPPLEMENTARY MATERIAL

## Data Availability

All data were provided in the supplementary material.

## Abbreviations

AI: Artificial intelligence
AUC: Area under hierarchical summary receiver operating characteristic curve
BCa: Bladder cancer
CI: Confidence interval
CLAIM: Checklist for Artificial Intelligence in Medical Imaging
CT: Computed tomography
DL: Deep learning
HSROC: Hierarchical summary receiver operating characteristic
MIBC: Muscle-invasive bladder cancer
MRI: Magnetic resonance imaging
NMIBC: Non-muscle-invasive bladder cancer
PC: Partial cystectomy
PROBAST: Prediction model Risk of Bias Assessment Tool
RC: Radical cystectomy
ROB: Risk of bias
RQS: Radiomics Quality Score
TURBT: Transurethral resection of bladder tumor
VI-RADS: Vesical Imaging-Reporting and Data System
