# Supplementary material for: The accuracy and quality of image-based artificial intelligence for muscle-invasive bladder cancer prediction
Source: Insights Imaging. 2024 Aug 1;15:185. doi: 10.1186/s13244-024-01780-y (PMC11294512; doi:10.1186/s13244-024-01780-y)
Supplement: Supplementary file 1 — ELECTRONIC SUPPLEMENTARY MATERIAL [file 13244_2024_1780_MOESM1_ESM.pdf]

The accuracy and quality of image-based artificial intelligence for muscle-invasive bladder cancer prediction

ELECTRONIC SUPPLEMENTARY MATERIAL

Supplementary Material 1.

Section and Topic	Item #	Checklist item	Location where item is reported
**TITLE**			
Title	1	Identify the report as a systematic review.	Page 1
**ABSTRACT**			
Abstract	2	See the PRISMA 2020 for Abstracts checklist.	Page 1
**INTRODUCTION**			
Rationale	3	Describe the rationale for the review in the context of existing knowledge.	Page 3
Objectives	4	Provide an explicit statement of the objective(s) or question(s) the review addresses.	Page 4
**METHODS**			
Eligibility criteria	5	Specify the inclusion and exclusion criteria for the review and how studies were grouped for the syntheses.	Page 4
Information sources	6	Specify all databases, registers, websites, organisations, reference lists and other sources searched or consulted to identify studies. Specify the date when each source was last searched or consulted.	Page 4
Search strategy	7	Present the full search strategies for all databases, registers and websites, including any filters and limits used.	ESM1
Selection process	8	Specify the methods used to decide whether a study met the inclusion criteria of the review, including how many reviewers screened each record and each report retrieved, whether they worked independently, and if applicable, details of automation tools used in the process.	Page 4
Data collection process	9	Specify the methods used to collect data from reports, including how many reviewers collected data from each report, whether they worked independently, any processes for obtaining or confirming data from study investigators, and if applicable, details of automation tools used in the process.	Page 4
Data items	10a	List and define all outcomes for which data were sought. Specify whether all results that were compatible with each outcome domain in each study were sought (e.g. for all measures, time points, analyses), and if not, the methods used to decide which results to collect.	Page 4
	10b	List and define all other variables for which data were sought (e.g. participant and intervention characteristics, funding sources). Describe any assumptions made about any missing or unclear information.	Page 5
Study risk of bias assessment	11	Specify the methods used to assess risk of bias in the included studies, including details of the tool(s) used, how many reviewers assessed each study and whether they worked independently, and if applicable, details of automation tools used in the process.	Page 5
Effect measures	12	Specify for each outcome the effect measure(s) (e.g. risk ratio, mean difference) used in the synthesis or presentation of results.	Page 5

Section and Topic	Item #	Checklist item	Location where item is reported
Synthesis methods	13a	Describe the processes used to decide which studies were eligible for each synthesis (e.g. tabulating the study intervention characteristics and comparing against the planned groups for each synthesis (item #5)).	Page 5
	13b	Describe any methods required to prepare the data for presentation or synthesis, such as handling of missing summary statistics, or data conversions.	Page 5-7
	13c	Describe any methods used to tabulate or visually display results of individual studies and syntheses.	Page 5, ESM 2
	13d	Describe any methods used to synthesize results and provide a rationale for the choice(s). If meta-analysis was performed, describe the model(s), method(s) to identify the presence and extent of statistical heterogeneity, and software package(s) used.	Page 6
	13e	Describe any methods used to explore possible causes of heterogeneity among study results (e.g. subgroup analysis, meta-regression).	Page 6
	13f	Describe any sensitivity analyses conducted to assess robustness of the synthesized results.	Page 6
Reporting bias assessment	14	Describe any methods used to assess risk of bias due to missing results in a synthesis (arising from reporting biases).	Page 5-6
Certainty assessment	15	Describe any methods used to assess certainty (or confidence) in the body of evidence for an outcome.	Page 6
**RESULTS**			
Study selection	16a	Describe the results of the search and selection process, from the number of records identified in the search to the number of studies included in the review, ideally using a flow diagram.	Page 4
	16b	Cite studies that might appear to meet the inclusion criteria, but which were excluded, and explain why they were excluded.	Page 5-7
Study characteristics	17	Cite each included study and present its characteristics.	Page 7, table
Risk of bias in studies	18	Present assessments of risk of bias for each included study.	Page 8-10, ESM 3
Results of individual studies	19	For all outcomes, present, for each study: (a) summary statistics for each group (where appropriate) and (b) an effect estimate and its precision (e.g. confidence/credible interval), ideally using structured tables or plots.	Page 10, ESM 2-3
Results of syntheses	20a	For each synthesis, briefly summarise the characteristics and risk of bias among contributing studies.	Page 8-10
	20b	Present results of all statistical syntheses conducted. If meta-analysis was done, present for each the summary estimate and its precision (e.g. confidence/credible interval) and measures of statistical heterogeneity. If comparing groups, describe the direction of the effect.	Page 10
	20c	Present results of all investigations of possible causes of heterogeneity among study results.	Page 10
	20d	Present results of all sensitivity analyses conducted to assess the robustness of the synthesized results.	Page 10
Reporting biases	21	Present assessments of risk of bias due to missing results (arising from reporting biases) for each synthesis assessed.	Page 10
Certainty of evidence	22	Present assessments of certainty (or confidence) in the body of evidence for each outcome assessed.	Page 8-12
**DISCUSSION**			
Discussion	23a	Provide a general interpretation of the results in the context of other evidence.	Page 11

Section and Topic	Item #	Checklist item	Location where item is reported
	23b	Discuss any limitations of the evidence included in the review.	Page 12
	23c	Discuss any limitations of the review processes used.	Page 12
	23d	Discuss implications of the results for practice, policy, and future research.	Page 12
**OTHER INFORMATION**			
Registration and protocol	24a	Provide registration information for the review, including register name and registration number, or state that the review was not registered.	International Prospective Register of Systematic Reviews (CRD42023446035)
	24b	Indicate where the review protocol can be accessed, or state that a protocol was not prepared.	Page 4
	24c	Describe and explain any amendments to information provided at registration or in the protocol.	Page 4
Support	25	Describe sources of financial or non-financial support for the review, and the role of the funders or sponsors in the review.	Declarations
Competing interests	26	Declare any competing interests of review authors.	Declarations
Availability of data, code and other materials	27	Report which of the following are publicly available and where they can be found: template data collection forms; data extracted from included studies; data used for all analyses; analytic code; any other materials used in the review.	Declarations

From: Page MJ, McKenzie JE, Bossuyt PM, Boutron I, Hoffmann TC, Mulrow CD, et al. The PRISMA 2020 statement: an updated guideline for reporting systematic reviews. BMJ 2021;372:n71. doi: 10.1136/bmj.n71

For more information, visit: http://www.prisma-statement.org/

Supplementary Material 2:

Appendix 1. Search queries

Step	Query
1	(artificial intelligence OR machine learning OR radiomic* OR deep learning).af
2	(bladder cancer OR bladder carcinoma OR urothelial carcinoma).af
3	(stage OR staging OR muscle invasi*).af
4	(computed tomography OR CT OR magnetic resonance imaging OR MRI OR ultrasound) .af
5	English.lg
6	1 AND 2 AND 3 AND 4 AND 5

Supplementary Figures

Figure S1. The ROC plot for CT in the prediction of muscle-invasive bladder cancer

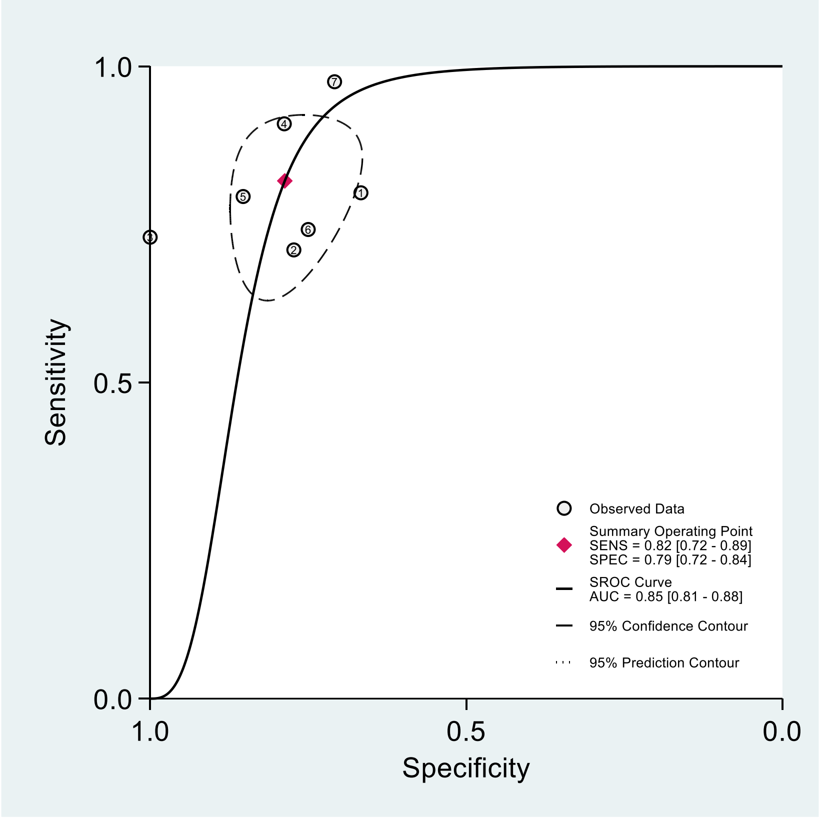

Figure S2. The forest plot for CT in the prediction of muscle-invasive bladder cancer

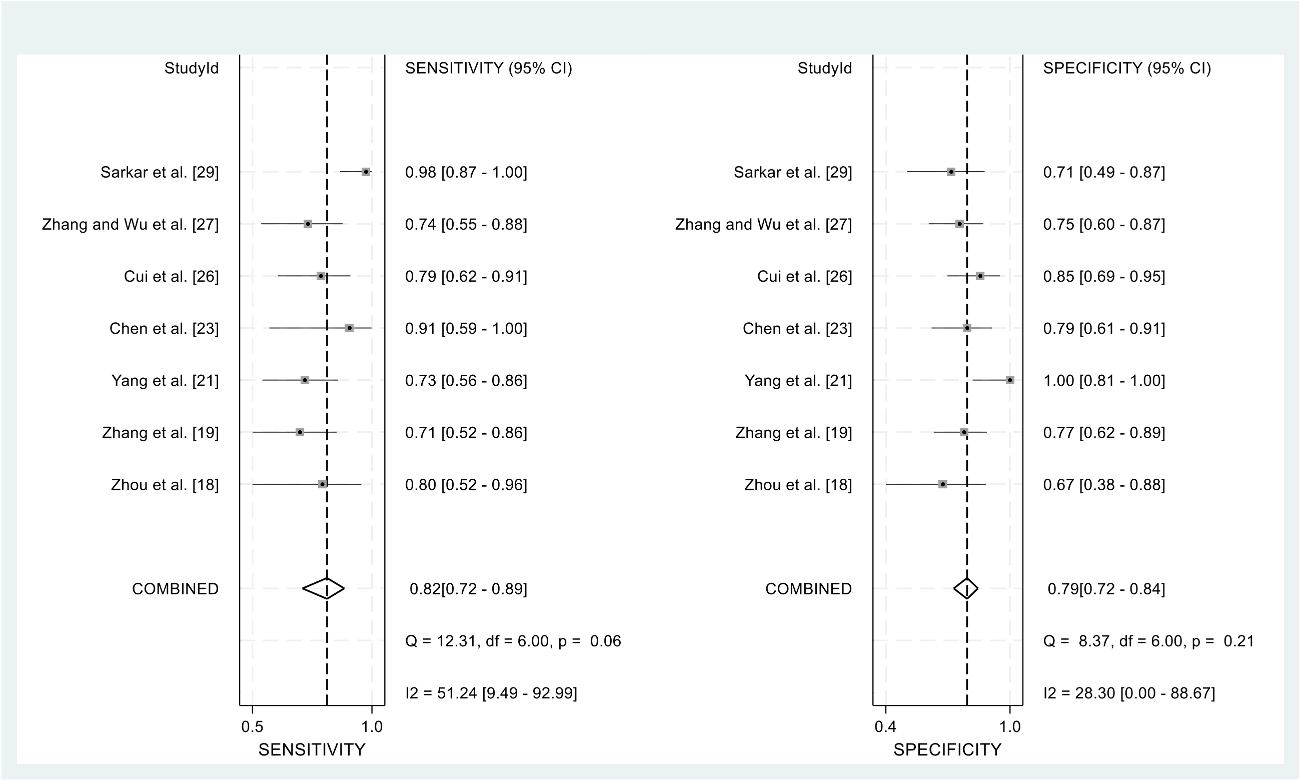

Figure S3. The ROC plot for MRI in the prediction of muscle-invasive bladder cancer

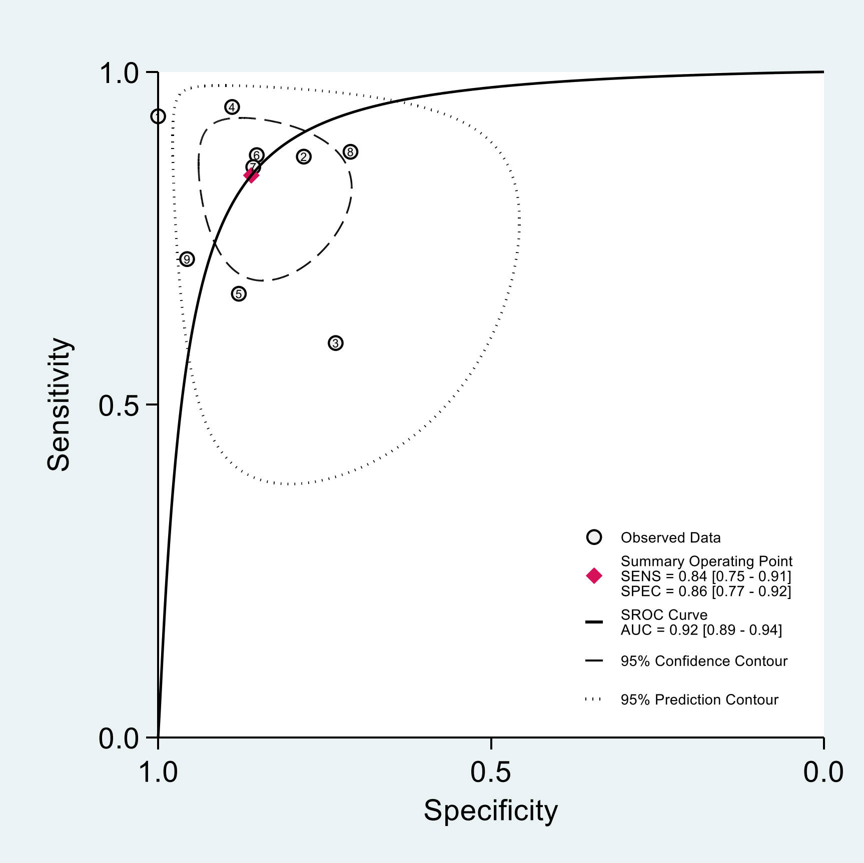

Figure S4. The forest plot for MRI in the prediction of muscle-invasive bladder cancer

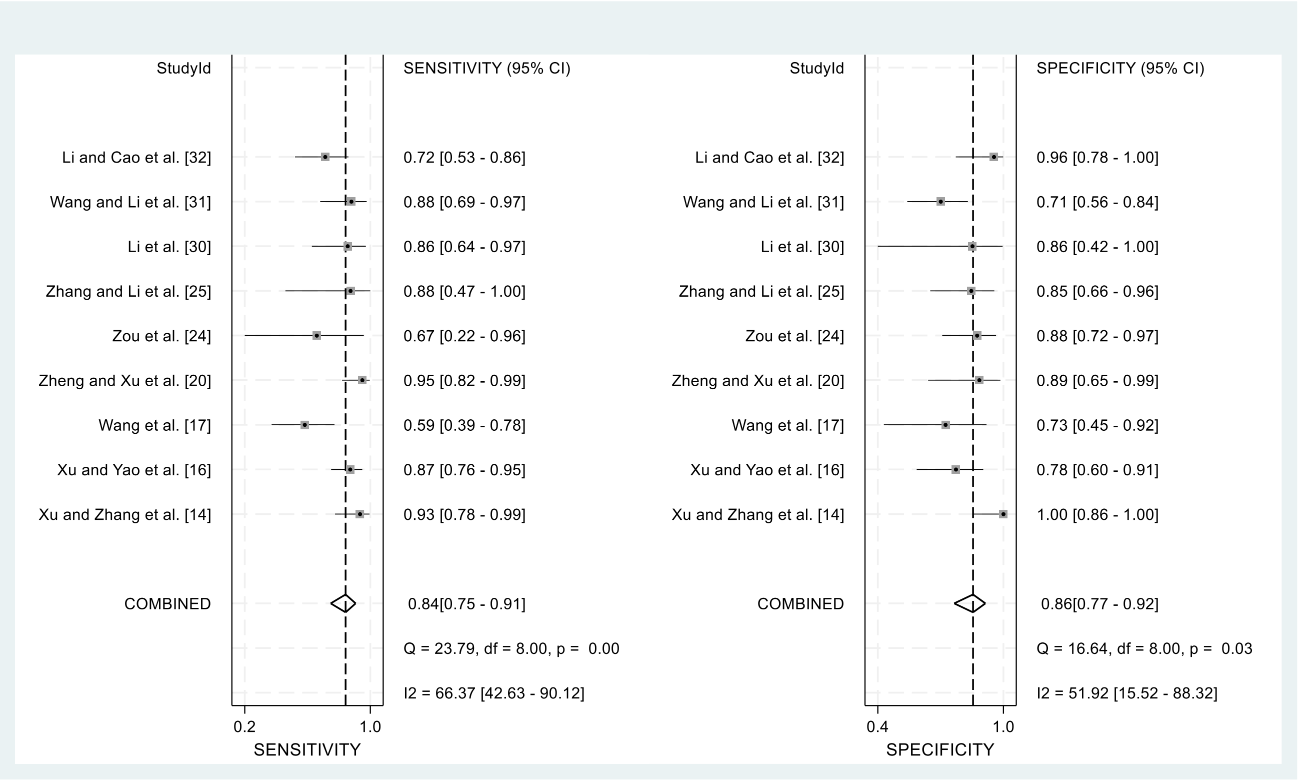

Figure S5. The ROC plot for radiomics in the prediction of muscle-invasive bladder cancer

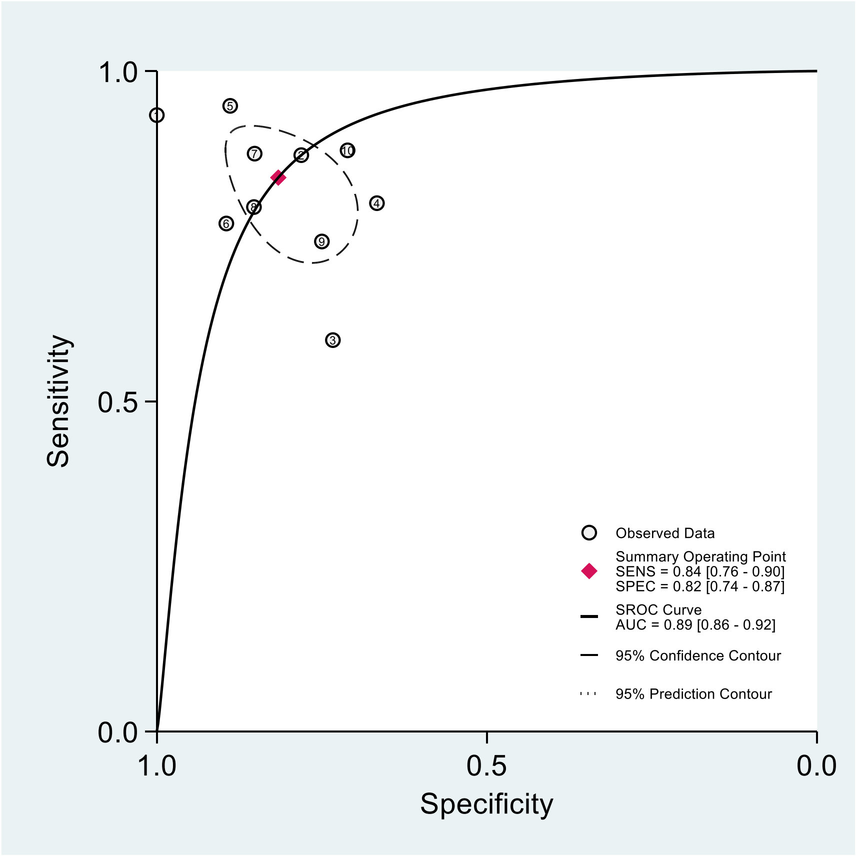

Figure S6. The forest plot for radiomics in the prediction of muscle-invasive bladder cancer

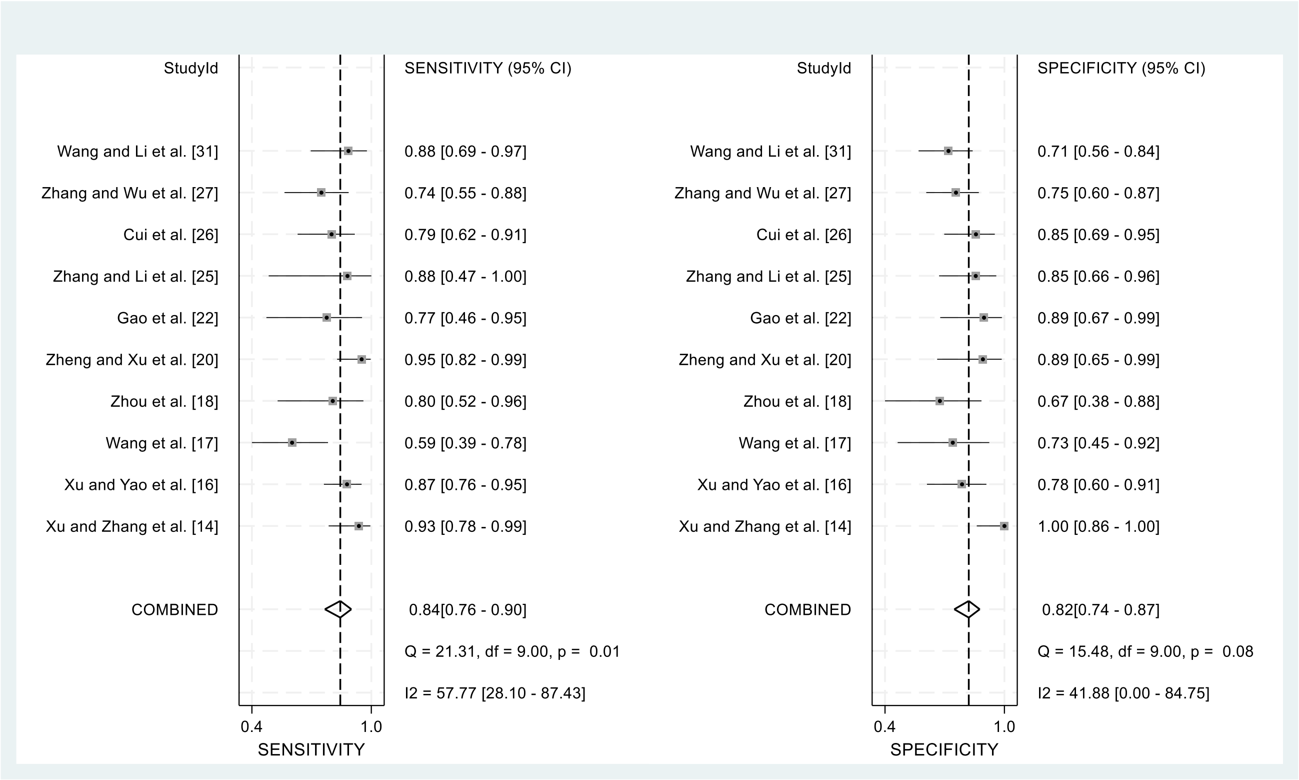

Figure S7. The ROC plot for deep learning in the prediction of muscle-invasive bladder cancer

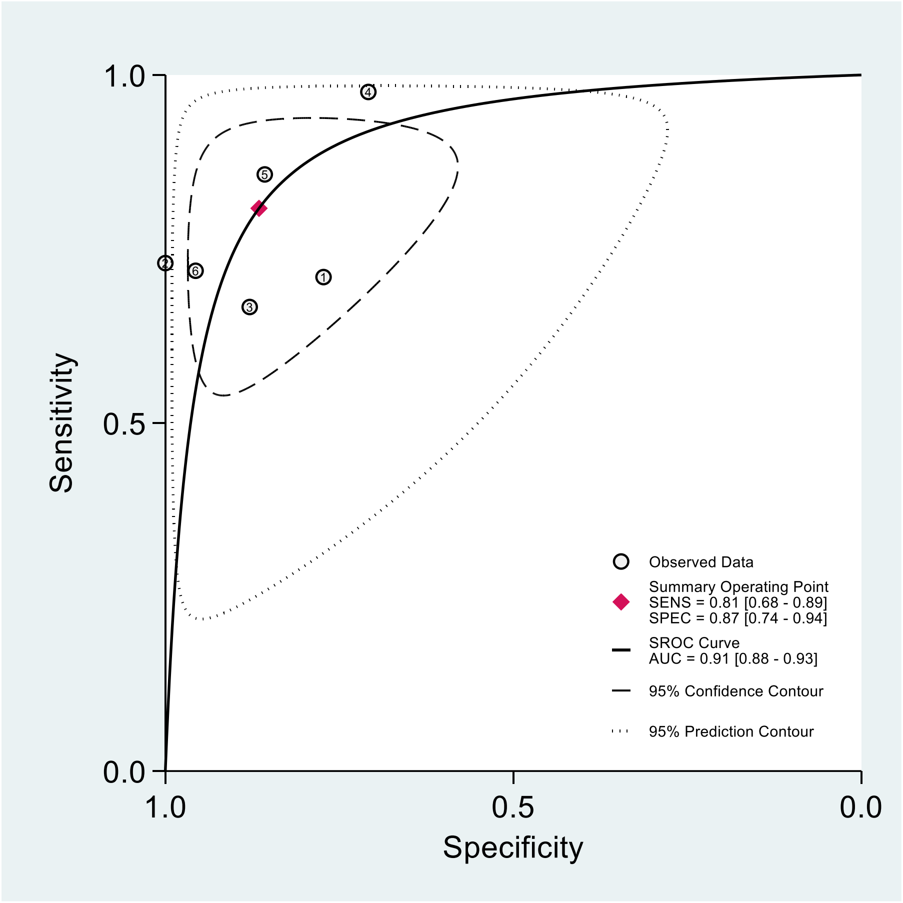

Figure S8. The forest plot for deep learning in the prediction of muscle-invasive bladder cancer

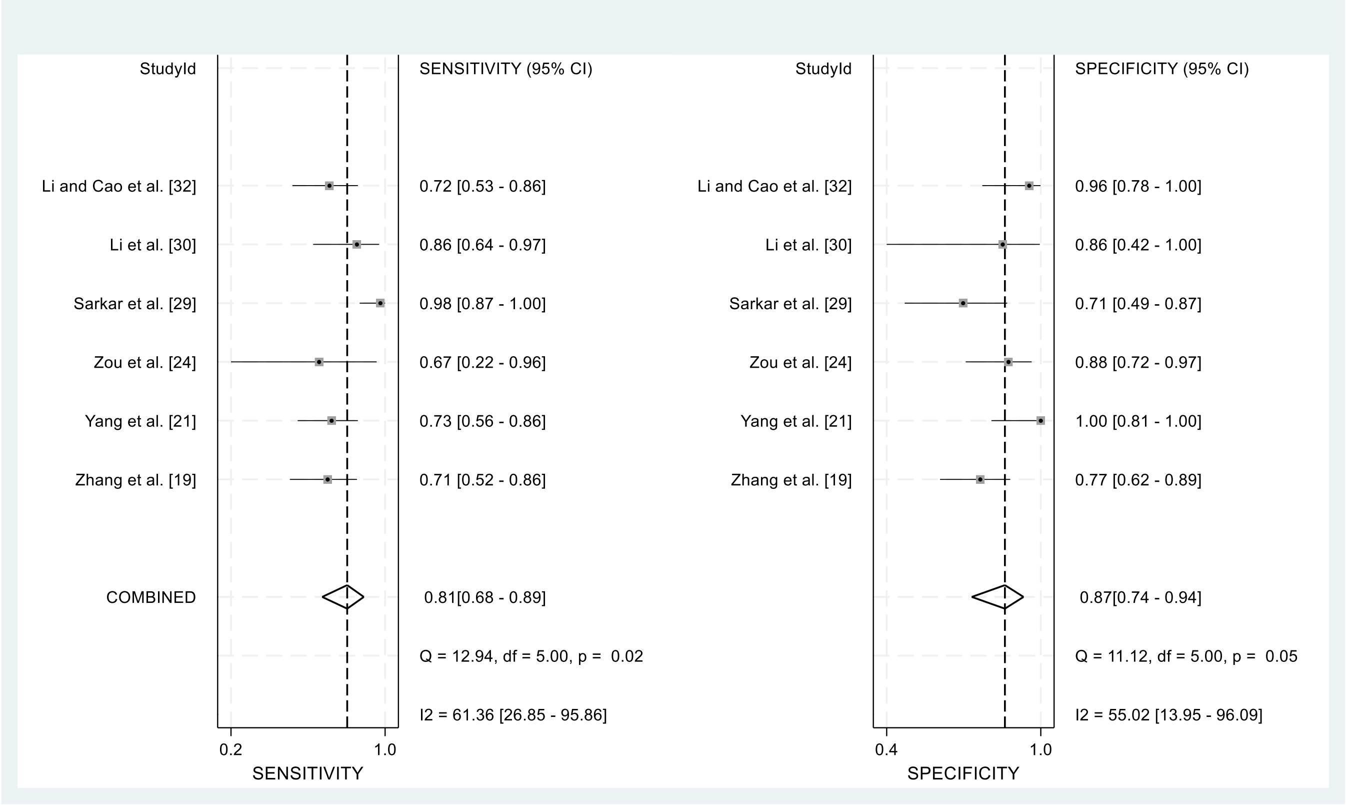

Supplementary Material 3:

Meta-analysis Raw Data:

citation	tp	fp	tn	fn	sen	spe	AUC	MIBC ratio	sample size
Xu and Zhang et a	28	0	24	2	0.926	1	0.9857	0.5556	54
Xu and Yao et al. [	48	7	25	7	0.873	0.781	0.907	0.6468	218
Wang et al. [17]	16	4	11	11	0.6	0.741	0.672	0.566	106
Zhou et al. [18]	12	5	10	3	0.8182	0.6842	0.782	0.5	100
Zhang et al. [19]	22	10	34	9	0.71	0.773	0.791	0.2766	441
Zheng and Xu et a	36	2	16	2	0.9444	0.8684	0.906	0.3351	185
Yang et al. [21]	27	0	18	10	0.722	1	0.998	0.3252	369
Gao et al. [22]	10	2	17	3	0.77	0.89	0.84	0.4135	104
Chen et al. [23]	10	7	26	1	0.909	0.788	0.884	0.2486	173
Zou et al. [24]	4	4	29	2	0.667	0.879	0.856	0.2863	468
Zhang and Li et al.	7	4	23	1	0.8909	0.8424	0.931	0.231	342
Cui et al. [26]	27	5	29	7	0.794	0.853	0.894	0.5	188
Zhang and Wu et a	23	11	33	8	0.742	0.75	0.784	0.2766	441
Sarkar et al. [29]	40	7	17	1	0.9675	0.6965	/	0.6307	65
Li et al. [30]	18	1	6	3	0.857	0.857	0.932	0.3305	121
Wang and Li et al.	22	13	32	3	0.88	0.711	0.711	0.3717	191
Li and Cao et al. [3	23	1	22	9	0.719	0.957	0.861	0.2884	215

CLAIM:

		item_nun	detail	Xu et al. [	Garapati	Xu and Z	Zheng et	Xu and Y	Wang et a	Zhou et a	Zhang et	Zheng an	Yang et a	Gao et al.	Chen et a	Zou et al.	Zhang an	Cui et al.	Zhang an	Liu et al.	Sarkar et	Li et al. [	Wang and	Li and Cao et al. [3		adherenc	total stud	adherenc
TITLE or		1	Identification	1	1	1	1	1	1	1	1	1	1	1	1	1	1	1	1	1	1	1	1	1		21	21	1
ABSTR		2	Structured su	1	1	1	1	1	1	1	1	1	1	1	1	1	1	1	1	1	0	1	1	1		20	21	0.95238
INTROD		3	Scientific and	1	1	1	1	1	1	1	1	1	1	1	1	1	1	1	1	1	1	1	1	1		21	21	1
UCTION		4	Study objecti	1	1	1	1	1	1	1	1	1	1	1	1	1	1	1	1	1	0	1	1	1		20	21	0.95238
	Study	5	Prospective c	1	1	1	1	1	1	1	1	1	1	1	1	1	1	1	1	1	1	1	1	1		21	21	1
	Design	6	Study goal, su	1	1	1	1	1	1	1	1	1	1	1	1	1	1	1	1	1	1	1	1	1		21	21	1
	Data	7	Data sources	1	1	1	1	1	1	1	1	1	1	1	1	1	1	1	1	1	1	1	1	1		21	21	1
		8	Eligibility crit	1	0	1	1	1	1	1	1	1	1	1	1	1	1	1	1	1	0	1	1	1		19	21	0.90476
		9	Data preproc	0	0	1	0	0	0	1	1	0	1	1	1	1	0	0	0	0	0	1	0	1		9	21	0.42857
		10	Selection of c	NA	NA	NA	NA	NA	NA	NA	NA	NA	NA	NA	NA	NA	NA	1	NA	NA	NA	NA	NA	NA		1	1	1
		11	Definitions of	1	1	1	1	1	1	1	1	1	1	1	1	1	1	1	1	1	1	1	1	1		21	21	1
		12	De-identifica	0	0	0	0	0	0	0	0	0	0	0	0	0	0	0	0	0	0	0	0	0		0	21	0
		13	How missing	0	0	0	0	0	0	1	0	0	0	1	0	0	0	0	0	0	0	0	0	0		2	21	0.09524
	Ground	14	Definition of	1	0	0	1	1	1	0	1	1	1	0	1	1	1	1	1	0	1	1	1	1		16	21	0.7619
	Truth	15	Rationale for	NA	0	0	0	1	0	0	0	0	NA	0	0	NA	NA	0	0	0	NA	0	0	0		1	16	0.0625
		16	Source of gro	0	0	0	0	1	0	0	0	0	0	0	0	0	0	0	0	0	0	0	0	0		1	21	0.04762
		17	Annotation to	0	0	0	0	0	0	0	0	0	0	0	0	0	0	0	0	0	0	0	0	0		0	21	0
METHO		18	Measuremen	0	0	0	0	0	0	0	0	0	0	0	0	0	0	0	0	0	0	0	0	0		0	21	0
DS	Data	19	Intended sam	0	0	0	0	0	0	0	0	0	0	0	0	0	0	0	0	0	0	0	0	0		0	21	0
	Partitions	20	How data we	1	1	1	1	1	1	1	1	1	1	1	1	1	1	1	1	1	1	1	1	1		21	21	1
		21	Level at whic	1	1	1	1	1	1	1	1	1	1	1	1	1	1	1	1	1	1	1	1	1		21	21	1
	Model	22	Detailed desc	1	1	1	1	1	1	1	0	1	1	1	1	1	1	1	1	1	1	1	1	1		20	21	0.95238
		23	Software libr	0	0	0	1	1	1	1	1	1	1	1	1	1	1	1	1	1	1	1	1	1		18	21	0.85714
		24	Initialization c	0	0	0	0	0	0	0	0	0	1	0	1	0	0	0	0	0	1	0	0	0		3	21	0.14286
	Training	25	Details of tra	1	1	1	1	1	1	1	1	1	1	1	1	1	1	1	1	1	1	1	1	1		21	21	1
		26	Method of se	1	1	1	1	1	1	1	1	1	1	1	1	1	1	1	1	1	1	1	1	1		21	21	1
		27	Ensembling t	NA	NA	NA	NA	NA	NA	NA	NA	NA	NA	NA	NA	NA	NA	NA	NA	NA	NA	NA	NA	NA		0	0	#DIV/0!
	Evaluatio	28	Metrics of m	1	1	1	1	1	1	1	1	1	1	1	1	1	1	1	1	1	1	1	1	1		21	21	1
	n	29	Statistical me	1	1	1	1	0	1	1	1	1	0	0	1	1	1	1	1	1	1	1	1	1		18	21	0.85714
		30	Robustness c	0	0	0	0	0	0	0	0	0	0	0	0	0	0	0	0	0	0	0	0	0		0	21	0
		31	Methods for	0	0	0	0	0	0	0	1	0	1	0	0	1	0	0	0	0	0	1	0	1		5	21	0.2381
		32	Validation or	0	0	0	1	0	1	0	1	0	0	0	0	1	0	0	1	0	0	1	0	1		7	21	0.33333
	Data	33	Flow of parti	0	0	1	1	0	1	1	1	1	0	1	1	0	0	1	0	1	0	1	1	1		13	21	0.61905
RESULT		34	Demographic	0	0	1	1	1	1	1	1	1	1	1	1	1	1	1	1	1	0	1	1	1		18	21	0.85714
S	Model	35	Performance	1	1	1	1	1	1	1	1	1	1	1	1	1	1	1	1	1	1	1	1	1		21	21	1
	performa	36	Estimates of	1	1	1	1	0	1	1	1	1	0	0	1	1	1	1	1	1	1	1	1	1		18	21	0.85714
	nce	37	Failure analy	0	1	0	0	0	0	0	0	0	0	0	0	1	0	0	0	0	0	0	0	1		3	21	0.14286
DISCUS		38	Study limitati	1	1	1	1	1	1	1	1	1	1	1	1	1	1	1	1	1	1	1	1	1		21	21	1
SION		39	Implications f	1	1	1	1	1	1	0	1	1	1	1	1	1	0	0	1	1	0	0	0	1		15	21	0.71429
OTHER		40	Registration r	0	1	1	1	1	1	1	1	1	1	1	1	1	1	1	1	0	1	1	1	1		19	21	0.90476
INFORM		41	Where the fu	0	0	0	0	0	0	0	0	0	0	0	0	0	0	0	0	0	0	0	0	0		0	21	0
ATION		42	Sources of fu	1	0	0	1	1	1	1	1	1	0	0	0	1	0	0	1	0	1	1	1	1		13	21	0.61905
			**adherence i**	**22**	**21**	**24**	**27**	**25**	**27**	**26**	**28**	**26**	**25**	**24**	**27**	**29**	**23**	**25**	**26**	**23**	**21**	**28**	**25**	**30**				
			**total items**	**39**	**40**	**40**	**40**	**40**	**40**	**40**	**40**	**40**	**39**	**40**	**40**	**39**	**39**	**41**	**40**	**40**	**39**	**40**	**40**	**40**				
			**adherence r**	**0.5641**	**0.525**	**0.6**	**0.675**	**0.625**	**0.675**	**0.65**	**0.7**	**0.65**	**0.64103**	**0.6**	**0.675**	**0.74359**	**0.58974**	**0.60976**	**0.65**	**0.575**	**0.53846**	**0.7**	**0.625**	**0.75**				

RQS:

		Xu et al.	Garapati	Xu and Z	Zheng et	Xu and Y	Wang et	Zhou et a	Zhang et	Zheng an	Yang et a	Gao et al.	Chen et a	Zou et al.	Zhang an	Cui et al.	Zhang an	Liu et al.	Sarkar et	Li et al. [	Wang and	Li and Cao et al. [3	mean poi	median p	mean per	median percentage per item
criterion 1	Image protocol quality - well-documented image protocols (f	1	0	1	1	1	1	0	1	1	1	1	1	1	1	1	1	1	0	1	1	1	**0.85714**	**1**	**0.42857**	**0.5**
	**checkpoint_1**	**1**	**0**	**1**	**1**	**1**	**1**	**0**	**1**	**1**	**1**	**1**	**1**	**1**	**1**	**1**	**1**	**1**	**0**	**1**	**1**	**1**				
criterion 2	Multiple segmentations - possible actions are: segmentation b	1	0	0	1	1	1	1	1	1	1	1	1	0	1	1	1	1	0	0	1	0	**0.71429**	**1**	**0.35714**	**0.5**
criterion 3	Phantom study on all scanners - detect inter-scanner differer	0	0	0	0	0	0	0	0	0	0	0	0	0	0	0	0	0	0	0	0	0	**0**	**0**	**0**	**0**
criterion 4	Imaging at multiple time points - collect images of individuals	0	0	0	0	0	0	0	0	0	0	0	0	0	0	0	0	0	0	0	0	0	**0**	**0**	**0**	**0**
	**checkpoint_2**	**1**	**0**	**0**	**1**	**1**	**1**	**1**	**1**	**1**	**1**	**1**	**1**	**0**	**1**	**1**	**1**	**1**	**0**	**0**	**1**	**0**				
criterion 5	Feature reduction or adjustment for multiple testing - decreas	3	3	3	3	3	3	3	0	3	0	3	3	0	3	3	3	3	3	3	3	0	**2.42857**	**3**	**1.21429**	**1.5**
criterion 6	Multivariable analysis with non radiomics features (for exam	0	0	0	1	1	1	1	0	1	0	0	0	0	1	0	0	0	0	0	1	0	**0.33333**	**0**	**0.16667**	**0**
criterion 7	Detect and discuss biological correlates - demonstration of p	0	0	0	0	0	0	0	0	0	0	1	0	0	0	0	0	0	0	0	0	0	**0.04762**	**0**	**0.02381**	**0**
criterion 8	Cut-off analyses - determine risk groups by either the media	0	0	0	1	0	1	0	1	1	0	0	0	0	1	1	1	0	0	0	1	1	**0.42857**	**0**	**0.21429**	**0**
criterion 9	Discrimination statistics - report discrimination statistics (for	1	2	2	2	1	1	1	1	1	1	1	1	1	1	1	2	2	0	2	1	1	**1.2381**	**1**	**0.61905**	**0.5**
criterion 1	Calibration statistics - report calibration statistics (for exampl	0	0	0	2	0	1	1	1	1	0	0	0	0	1	0	1	0	0	0	0	1	**0.42857**	**0**	**0.21429**	**0**
criterion 1	Prospective study registered in a trial database - provides the	0	0	0	0	0	0	0	0	0	0	0	0	7	0	0	0	0	0	0	0	0	**0.33333**	**0**	**0.16667**	**0**
criterion 1	Validation - the validation is performed without retraining and	-5	2	2	3	2	3	2	3	2	2	2	2	4	2	2	3	2	2	3	2	3	**2.04762**	**2**	**1.02381**	**1**
criterion 1	Comparison to 'gold standard' - assess the extent to which th	0	0	0	2	2	2	0	2	2	0	0	0	0	0	2	0	2	0	0	2	2	**0.85714**	**0**	**0.42857**	**0**
criterion 1	Potential clinical utility - report on the current and potential a	0	0	0	2	0	2	2	2	2	0	0	0	0	2	2	0	0	0	0	0	2	**0.7619**	**0**	**0.38095**	**0**
criterion 1	Cost-effectiveness analysis - report on the cost-effectivenes	0	0	0	0	0	0	0	0	0	0	0	0	0	0	0	0	0	0	0	0	0	**0**	**0**	**0**	**0**
criterion 1	Open science and data - make code and data publicly availab	0	0	0	0	0	0	0	0	0	0	0	0	0	0	0	0	0	0	0	0	0	**0**	**0**	**0**	**0**
	**checkpoint_3**	**-1**	**7**	**7**	**16**	**9**	**14**	**10**	**10**	**13**	**3**	**7**	**6**	**12**	**11**	**11**	**10**	**9**	**5**	**8**	**10**	**10**				
	**total points**	**1**	**7**	**8**	**18**	**11**	**16**	**11**	**12**	**15**	**5**	**9**	**8**	**13**	**13**	**13**	**12**	**11**	**5**	**9**	**12**	**11**				
	**total percentage (n/36)**	**0.02778**	**0.19444**	**0.22222**	**0.5**	**0.30556**	**0.44444**	**0.30556**	**0.33333**	**0.41667**	**0.13889**	**0.25**	**0.22222**	**0.36111**	**0.36111**	**0.36111**	**0.33333**	**0.30556**	**0.13889**	**0.25**	**0.33333**	**0.30556**				

RQS Checklist:

		Points and Interpretation
criterion 1	Image protocol quality - well-documented image protocols (for example, contrast, slice thickness, energy, etc.) and/or usage of public image protocols allow reproducibility/replicability	+ 1 if protocols are well-documented<br>+ 1 if public protocol is used
	**checkpoint_1**	
criterion 2	Multiple segmentations - possible actions are: segmentation by different physicians/algorithms/software, perturbing segmentations by (random) noise, segmentation at different breathing cycles. Analyse feature robustness to segmentation variabilities	+ 1 if segmented multiple times (diferent physicians, algorithms, or perturbation of regions of interest)
criterion 3	Phantom study on all scanners - detect inter-scanner differences and vendor-dependent features. Analyse feature robustness to these sources of variability	+ 1 if texture phantoms were used for feature robustness assessment
criterion 4	Imaging at multiple time points - collect images of individuals at additional time points. Analyse feature robustness to temporal variabilities (for example, organ movement, organ expansion/shrinkage)	+ 1 multiple time points for feature robustness assessment
	**checkpoint_2**	
criterion 5	Feature reduction or adjustment for multiple testing - decreases the risk of overfitting. Overfitting is inevitable if the number of features exceeds the number of samples. Consider feature robustness when selecting features	- 3 if neither measure is implemented<br>+ 3 if either measure is implemented
criterion 6	Multivariable analysis with non radiomics features (for example, EGFR mutation) - is expected to provide a more holistic model. Permits correlating/inferencing between radiomics and non radiomics features	+ 1 if multivariable analysis with nonradiomics features
criterion 7	Detect and discuss biological correlates - demonstration of phenotypic differences (possibly associated with underlying gene–protein expression patterns) deepens understanding of radiomics and biology	+ 1 if present
criterion 8	Cut-off analyses - determine risk groups by either the median, a previously published cut-off or report a continuous risk variable. Reduces the risk of reporting overly optimistic results	+ 1 if cutoff either pre-defined or at median or continuous risk variable reported
criterion 9	Discrimination statistics - report discrimination statistics (for example, C-statistic, ROC curve, AUC) and their statistical significance (for example, p-values, confidence intervals). One can also apply resampling method (for example, bootstrapping, cross-validation)	+ 1 if a discrimination statistic and its statistical significance are reported<br>+ 1 if a resampling method technique is also applied
criterion 1[illegible]	Calibration statistics - report calibration statistics (for example, Calibration-in-the-large/slope, calibration plots) and their statistical significance (for example, P-values, confidence intervals). One can also apply resampling method (for example, bootstrapping, cross-validation)	+ 1 if a calibration statistic and its statistical significance are reported<br>+ 1 if a resampling method technique is also applied
criterion 1	Prospective study registered in a trial database - provides the highest level of evidence supporting the clinical validity and usefulness of the radiomics biomarker	+ 7 for prospective validation of a radiomics signature in an appropriate trial
criterion 1[illegible]	Validation - the validation is performed without retraining and without adaptation of the cut-off value, provides crucial information with regard to credible clinical performance	-5 if validation is missing<br>+ 2 if validation is based on a dataset from the same institute/<br>+ 3 if validation is based on a dataset from another institute/<br>+ 4 if validation is based on two datasets from two distinct institutes/<br>+4 if the study validates a previously published signature/<br>+5 if validation is based on three or more datasets from distinct institutes<br>*Datasets should be of comparable size and should have at least 10 events per model feature
criterion 1[illegible]	Comparison to 'gold standard' - assess the extent to which the model agrees with/is superior to the current 'gold standard' method (for example, TNM-staging for survival prediction). This comparison shows the added value of radiomics	+ 2 for comparison to gold standard
criterion 1[illegible]	Potential clinical utility - report on the current and potential application of the model in a clinical setting (for example, decision curve analysis).	+ 2 for reporting potential clinical utility
criterion 1[illegible]	Cost-effectiveness analysis - report on the cost-effectiveness of the clinical application (for example, QALYs generated)	+ 1 for cost-effectiveness analysis
criterion 1[illegible]	Open science and data - make code and data publicly available. Open science facilitates knowledge transfer and reproducibility of the study	+ 1 if scans are open source<br>+ 1 if region of interest segmentations are open source<br>+ 1 if code is open source<br>+ 1 if radiomics features are calculated on a set of representative ROIs and the calculated features and representative ROIs are open source
	**checkpoint_3**	
	**total points**	**Total points (36 = 100%)**
	**total percentage (n/36)**	
	Extracted from Lambin P, Leijenaar RTH, Deist TM, et al. Radiomics: the bridge between medical imaging and personalized medicine. Nat Rev Clin Oncol.<br>2017;14(12):749-762.	

Insights Imaging (2024) He C, Xu H, Yuan E, et al.

PROBAST:

			Xu et al. [	Garapati	Xu and Z	Zheng et	Xu and Y	Wang et a	Zhou et a	Zhang et	Zheng an	Yang et a	Gao et al.	Chen et a	Zou et al.	Zhang an	Cui et al.	Zhang an	Liu et al.	Sarkar et	Li et al. [	Wang and	Li and Cao et al. [3	number o	number o	number of no information
DOMAIN 1: Participants	A. Risk of	1.1 Were appropriate data sou	Y	Y	Y	Y	Y	Y	Y	Y	Y	Y	Y	Y	Y	Y	Y	Y	Y	Y	Y	Y	Y	21	0	0
		1.2 Were all inclusions and exc	Y	NI	Y	Y	Y	Y	Y	Y	Y	Y	Y	Y	Y	Y	Y	Y	Y	NI	Y	Y	Y	19	0	2
		**number of yes/probably yes**	**2**	**1**	**2**	**2**	**2**	**2**	**2**	**2**	**2**	**2**	**2**	**2**	**2**	**2**	**2**	**2**	**2**	**1**	**2**	**2**	**2**			
		**number of no/probably no**	**0**	**0**	**0**	**0**	**0**	**0**	**0**	**0**	**0**	**0**	**0**	**0**	**0**	**0**	**0**	**0**	**0**	**0**	**0**	**0**	**0**			
		**number of no information**	**0**	**1**	**0**	**0**	**0**	**0**	**0**	**0**	**0**	**0**	**0**	**0**	**0**	**0**	**0**	**0**	**0**	**1**	**0**	**0**	**0**			
		**Risk of bias introduced by s**	**low**	**unclear**	**low**	**low**	**low**	**low**	**low**	**low**	**low**	**low**	**low**	**low**	**low**	**low**	**low**	**low**	**low**	**unclear**	**low**	**low**	**low**			
DOMAIN 2: Predictors	A. Risk of Bias: List and	2.1 Were predictors defined an	Y	Y	NI	Y	Y	Y	Y	Y	Y	Y	Y	Y	Y	Y	Y	Y	PN	PN	Y	Y	Y	18	2	1
		2.2 Were predictor assessment	Y	Y	NI	NI	NI	Y	NI	Y	NI	NI	Y	NI	N	Y	N	Y	NI	NI	NI	NI	NI	7	2	12
		2.3 Are all predictors available	Y	Y	Y	Y	Y	Y	Y	Y	Y	Y	Y	Y	Y	Y	Y	Y	Y	Y	Y	Y	Y	21	0	0
		**number of yes/probably yes**	**3**	**3**	**1**	**2**	**2**	**3**	**2**	**3**	**2**	**2**	**3**	**2**	**2**	**3**	**2**	**3**	**1**	**1**	**2**	**2**	**2**			
		**number of no/probably no**	**0**	**0**	**0**	**0**	**0**	**0**	**0**	**0**	**0**	**0**	**0**	**0**	**1**	**0**	**1**	**0**	**1**	**1**	**0**	**0**	**0**			
		**number of no information**	**0**	**0**	**2**	**1**	**1**	**0**	**1**	**0**	**1**	**1**	**0**	**1**	**0**	**0**	**0**	**0**	**1**	**1**	**1**	**1**	**1**			
		**Risk of bias introduced by p**	**low**	**low**	**unclear**	**unclear**	**unclear**	**low**	**unclear**	**low**	**unclear**	**unclear**	**low**	**unclear**	**high**	**low**	**high**	**low**	**high**	**high**	**unclear**	**unclear**	**unclear**			
DOMAIN 3: Outcome	A. Risk of Bias: Describe the outcome, how it	3.1 Was the outcome determin	PY	NI	PY	PY	Y	PY	PY	PY	PY	PY	NI	PY	PY	PY	PY	PY	NI	NI	PY	PY	PY	17	0	4
		3.2 Was a prespecified or stan	NI	NI	NI	NI	Y	NI	NI	NI	NI	NI	NI	NI	NI	NI	NI	NI	NI	NI	NI	NI	NI	1	0	20
		3.3 Were predictors excluded f	Y	Y	Y	Y	Y	Y	Y	Y	Y	Y	Y	Y	Y	Y	Y	Y	Y	Y	Y	Y	Y	21	0	0
		3.4 Was the outcome defined a	NI	NI	NI	NI	PY	NI	NI	NI	NI	NI	NI	NI	NI	NI	NI	NI	NI	NI	NI	NI	NI	1	0	20
		3.5 Was the outcome determin	NI	NI	NI	NI	PY	NI	NI	NI	NI	NI	NI	NI	NI	NI	NI	NI	NI	NI	NI	NI	NI	1	0	20
		3.6 Was the time interval betw	Y	NI	Y	Y	Y	Y	Y	Y	Y	Y	NI	Y	Y	Y	Y	Y	NI	NI	Y	Y	Y	17	0	4
		**number of yes/probably yes**	**3**	**1**	**3**	**3**	**6**	**3**	**3**	**3**	**3**	**3**	**1**	**3**	**3**	**3**	**3**	**3**	**1**	**1**	**3**	**3**	**3**			
		**number of no/probably no**	**0**	**0**	**0**	**0**	**0**	**0**	**0**	**0**	**0**	**0**	**0**	**0**	**0**	**0**	**0**	**0**	**0**	**0**	**0**	**0**	**0**			
		**number of no information**	**3**	**5**	**3**	**3**	**0**	**3**	**3**	**3**	**3**	**3**	**5**	**3**	**3**	**3**	**3**	**3**	**5**	**5**	**3**	**3**	**3**			
		**Risk of bias introduced by t**	**unclear**	**unclear**	**unclear**	**unclear**	**low**	**unclear**	**unclear**	**unclear**	**unclear**	**unclear**	**unclear**	**unclear**	**unclear**	**unclear**	**unclear**	**unclear**	**unclear**	**unclear**	**unclear**	**unclear**	**unclear**			
DOMAIN 4: Analysis	Risk of Bias:Describe numbers of participants, number of	4.1 Were there a reasonable n	N	N	N	N	N	N	N	NI	N	PY	N	N	N	N	N	N	N	N	N	N	N	1	19	1
		4.2 Were continuous and categ	Y	Y	Y	Y	Y	Y	Y	Y	Y	Y	Y	Y	Y	Y	Y	Y	Y	Y	Y	Y	Y	21	0	0
		4.3 Were all enrolled participar	Y	Y	Y	Y	Y	Y	Y	Y	Y	Y	Y	Y	Y	Y	N	Y	Y	Y	Y	Y	Y	20	1	0
		4.4 Were participants with mis	NI	NI	NI	NI	NI	NI	Y	NI	NI	NI	Y	NI	NI	NI	NI	NI	NI	NI	NI	NI	NI	2	0	19
		4.5 Was selection of predictors	N	Y	Y	Y	Y	Y	Y	Y	N	Y	N	Y	Y	Y	N	Y	Y	Y	Y	N	Y	16	5	0
		4.6 Were complexities in the da	Y	Y	Y	Y	Y	Y	Y	Y	Y	Y	Y	Y	Y	Y	Y	Y	Y	Y	Y	Y	Y	21	0	0
		4.7 Were relevant model perfo	N	N	N	Y	N	Y	Y	Y	Y	N	N	N	N	Y	N	Y	N	N	N	N	Y	8	13	0
		4.8 Were model overfitting, und	N	Y	Y	Y	N	N	N	N	N	N	N	N	N	N	N	Y	Y	Y	Y	N	N	7	14	0
		4.9 Do predictors and their ass	Y	Y	Y	Y	Y	Y	Y	Y	Y	Y	Y	Y	Y	Y	Y	Y	Y	Y	Y	Y	Y	21	0	0
		**number of yes/probably yes**	**4**	**6**	**6**	**7**	**5**	**6**	**7**	**6**	**5**	**6**	**5**	**5**	**5**	**6**	**3**	**7**	**6**	**6**	**6**	**4**	**6**			
		**number of no/probably no**	**4**	**2**	**2**	**1**	**3**	**2**	**2**	**1**	**3**	**2**	**4**	**3**	**3**	**2**	**5**	**1**	**2**	**2**	**2**	**4**	**2**			
		**number of no information**	**1**	**1**	**1**	**1**	**1**	**1**	**0**	**2**	**1**	**1**	**0**	**1**	**1**	**1**	**1**	**1**	**1**	**1**	**1**	**1**	**1**			
		**Risk of bias introduced by t**	**high**	**high**	**high**	**high**	**high**	**high**	**high**	**high**	**high**	**high**	**high**	**high**	**high**	**high**	**high**	**high**	**high**	**high**	**high**	**high**	**high**			
		**total number of yes/probably**	**12**	**11**	**12**	**14**	**15**	**14**	**14**	**14**	**12**	**13**	**11**	**12**	**12**	**14**	**10**	**15**	**10**	**9**	**13**	**11**	**13**			
		**total number of no/probably**	**4**	**2**	**2**	**1**	**3**	**2**	**2**	**1**	**3**	**2**	**4**	**3**	**4**	**2**	**6**	**1**	**3**	**3**	**2**	**4**	**2**			
		**total number of no informati**	**4**	**7**	**6**	**5**	**2**	**4**	**4**	**5**	**5**	**5**	**5**	**5**	**4**	**4**	**4**	**4**	**7**	**8**	**5**	**5**	**5**			
Overall judgement of risk		**total Risk of bias**	**high**	**high**	**high**	**high**	**high**	**high**	**high**	**high**	**high**	**high**	**high**	**high**	**high**	**high**	**high**	**high**	**high**	**high**	**high**	**high**	**high**			
